# Systematic literature review evaluating evidence and mechanisms of action for platelet-rich plasma as an antibacterial agent

**DOI:** 10.1186/s13019-021-01652-2

**Published:** 2021-09-28

**Authors:** Dalip Sethi, Kimberly E. Martin, Sangeeta Shrotriya, Bethany L. Brown

**Affiliations:** 1 Terumo Blood and Cell Technologies, Inc., 10810 West Collins Avenue, Lakewood, CO 80215 USA; 2 Boulder Clinical Science, 302 Urban Prairie St., Fort Collins, CO 80524 USA; 3 Servier Pharmaceuticals, 200 Pier 4, Boston, MA 02210 USA; 4 American Red Cross, Biomedical Services, Holland Laboratory, Rockville, MD 20855 USA

**Keywords:** Platelet rich plasma, Wound infection, Deep sternal wound infection, Systematic literature search, Review

## Abstract

Platelet rich plasma or PRP is a supraphysiologic concentrate of platelets derived by centrifugation and separation of whole blood components. Along with platelets and plasma, PRP contains various cell types including white blood cells (WBC)/leukocytes, both granulocytes (neutrophils, basophils, eosinophils) and agranulocytes (monocytes, lymphocytes). Researchers and clinicians have explored the application of PRP in wound healing and prevention of surgical wound infections, such as deep sternal wounds. We conducted this systematic literature review to evaluate the preclinical and clinical evidence for the antibacterial effect of PRP and its potential mechanism of action. 526 records were identified for screening. 34 unique articles were identified to be included in this literature review for data summary. Overall, the quality of the clinical trials in this review is low, and collectively qualify as Oxford level C. Based on the available clinical data, there is a clear trend towards safety of autologous PRP and potential efficacy in deep sternal wound management. The preclinical and bench data is very compelling. The application of PRP in treatment of wounds or prevention of infection with PRP is promising but there is a need for foundational bench and preclinical animal research to optimize PRP as an antibacterial agent, and to provide data to aid in the design and conduct of well-designed RCTs with adequate power to confirm antimicrobial efficacy of PRP in specific disease states and wound types.

## Scope of the systematic literature review

### Objective

The objective of this systematic literature review is to evaluate the preclinical and clinical evidence for the antibacterial effect of the Platelet-Rich Plasma (PRP) in wound healing, with a focus on its application in prevention of deep sternal wound infections (DSWI), and to generate testable hypotheses for the mechanism of action

### Description of the PRP concentration systems in scope for the systematic literature review

The PRP is prepared using a whole blood centrifugation, platelet concentration system. The system generally consists of an automated, dedicated centrifuge and accessory processing disposables that separates whole blood into an autologous platelet concentrate. The centrifuge is automated and easy to operate with minimal training. The unit is portable and small enough to remain in the procedure room, which allows collection, processing, and delivery to occur during the same patient visit. Whole blood is typically collected by venipuncture and injected into a sterile disposable dual centrifuge tube along with an anticoagulant. The centrifuge spins automatically through a 2-step process. After the automated centrifuge cycle, red blood cells (RBCs) are found in one chamber and the collected platelets are found at the bottom of the second chamber in a platelet-poor plasma (PPP) supernatant. The platelet-poor fraction of the plasma in the second chamber is aspirated with a sterile syringe, leaving a volume sufficient to resuspend the collected platelets at the desired concentration. The resulting PRP can be collected in a blunt-tipped, sterile syringe and is ready for the addition of thrombin for activation prior to application to the surgical site. The PRP Concentration System subjects the platelets to minimal manipulation by centrifugation and the cells are never removed from the plasma environment.

### Introduction

Autologous PRP is a concentrate of platelets derived from the patient’s own blood. PRP has a supraphysiologic platelet concentration approximately 3 to 5 times above what is found in whole blood that stimulates clotting and may play a role in wound healing. In recent years PRP has gained attention due to its potential in regenerative medicine, including cardiovascular surgery, soft tissue repair (eg, ligament, tendon, muscle), dermatology, urology, orthopedics, cosmetics, and faciomaxillary surgery [1, 2]. Clinical studies have shown promise of platelet concentrate application in soft tissue healing while other studies also have suggested benefits in oral and maxillofacial surgery as PRP enhanced bone grafts [3–6].

PRP contains various cell types including white blood cells (WBC)/leukocytes, both granulocytes (neutrophils, basophils, eosinophils) and agranulocytes (monocytes, lymphocytes), and peripheral progenitor cells; however, the major component is platelets [7–9]. Activated PRP releases a number of growth factors: platelet-derived growth factor (PDGF), vascular epithelial growth factor (VEGF), transforming growth factors (TGF-β1 and TGF-β2), epithelial growth factor (EGF), and insulin-like growth factor (IGF) through autocrine (influencing its own cell membrane) and paracrine (influencing another cell membrane) mechanisms [8, 9]. Moreover, PRP also includes immune system messengers, enzymes and their inhibitors, and plasma complement that have been suggested to participate in bacteria control, tissue repair, and wound healing [7, 10–12]. PRP has been shown to influence the migration, proliferation, and differentiation of several cell types although the exact mechanism involved in this process is largely unknown. Many PRP preparations contain a greater number of leukocytes than whole blood. There have been reports that PRP processing concentrates leukocytes by 5- to tenfold although the beneficial role of leukocytes in PRP is debated in the literature. Some studies have reported that increased concentration of leukocytes may improve recruitment of immune cells, stabilize the matrix, and regulate the inflammatory response [13, 14]. In contrast, other studies suggest that increased leukocytes might increase the inflammatory response by secreting various proinflammatory proteases, which might delay the healing process [13, 15].

Wound healing is a coordinated dynamic tissue repair process that involves the interaction of multiple cell types, growth factors, cytokines, and chemokines that can be influenced by various pathophysiological factors and exogenous factors (microorganisms) [16–18]. The continued presence of bacteria at the wound site produces inflammatory mediators that hinder the wound healing process [17]. The bacterial screening of acute wounds (eg, primarily abscesses, surgical, and traumatic wounds) and chronic wounds (eg, leg ulcers) have revealed that diverse gram-positive or gram-negative bacteria can colonize the wound site, either singularly or in a polymicrobial infection [17, 19]. The samples isolated from acute wounds have shown that *Staphylococcus aureus* (*S. aureus*) was the most prevalent bacteria, and frequently existed as a pure population or in combination with other gram-positive aerobes [17, 19]. Chronic wounds display a combination of aerobic-anaerobic microflora. Aerobic pathogens (including facultative), most notably *S. aureus*, *Escherichia coli* (*E. coli*), and *Pseudomonas aeruginosa* (*P. aeruginosa*), have frequently been associated with delayed healing and infection in both acute and chronic wounds [17]. Host defense mechanisms, patient comorbid conditions, such as diabetes, and overall host status play a large role in infections and wound-delayed healing. In general, wound management is a multifaced approach that includes controlling bacterial growth and inflammation, maintaining adequate tissue perfusion, and restoring the damaged tissue. In addition to these complexities in wound management, emergence of antimicrobial resistance may also impact morbidity and mortality. Hence, there is significant interest to develop novel strategies to improve bacterial control and wound healing for prevention and treatment of acute and chronic wounds.

In recent years, autologous PRP has emerged as a potential option to prevent or treat postoperative acute infections, chronic wound infections, or osteomyelitis [4, 12, 20]. There has been promising data specifically related to prevention of deep sternal wound infection (DSWI) [4]. The concept of using PRP for its antibacterial effect is not new and dates back several decades [8, 13]. Platelets are reported to have multiple functions that integrate innate and adaptive antibacterial host defenses [7, 10]. Some studies have reported that platelets actively sense signals from the site of injury and microbial threats, express a wide range of antibacterial proteins and potential bacterial receptors, release a broad variety of molecules that alter host defense mechanism, and develop the ability to internalize bacteria [7, 10, 13]. Various studies have demonstrated that, once activated, platelets are able to release antimicrobial peptides or kinocidins (such as CXCL4, CXCL7, CXCL5) with activity against bacteria and fungi [11, 21–23]. Substantial efforts have been made to isolate, characterize, and study the role of specific antimicrobial molecules from platelets of human and animal origin with limited success [7, 21]. Platelets are also suggested to generate reactive oxygen species (ROS) that can bind and internalize microorganisms and participate in antibody-dependent cellular cytotoxicity [24, 25]. Recent studies highlighted the direct role of platelets in identifying, sequestering, and counteracting invading pathogens, as well as their role in recruiting leukocytes to infection sites, further enhancing their ability to phagocytose and kill microorganisms by triggering different types of signaling pathways [7]. PRP has been extensively studied but defining an optimal preparation method and defining dosing in relation to patient host status or in relation to intended therapeutic effect have not been achieved. In addition, the specific role of each of the components, including leukocytes, growth factors, plasma components, and the possible synergistic effect of these components that might contribute to prevent the bacterial growth and restore damaged tissue is poorly understood.

With prevention of DSWI as the focus, this systematic review was conducted to answer the following research questions: a) does PRP exert antibacterial effect?; b) is the effect of PRP bactericidal or bacteriostatic?; c) which types of bacteria are affected by PRP?; d) what is the mechanism involved in the antibacterial effect of different components of PRP?; e) is there a way to enhance the antibacterial effect of PRP?; and f) is there any synergistic effect of different agents when used together? Based on these questions, the main objective of this systematic literature review was to explore the potential mechanism behind the antibacterial effect of platelet preparations based on the available preclinical and clinical evidence.1.4 Rationale for performing the systematic literature review.

Autologous PRP gel (PRG or PLG or PG) consists of various cellular components, cytokines, antimicrobial proteins, growth factors, chemokines, immune mediators, and a fibrin scaffold derived from a patient's blood. In recent years, PRP has gained popularity due to its potential to stimulate and accelerate the wound healing process. PRP has been shown to exert beneficial effects by relieving postsurgical discomfort and preventing infection in some studies, suggesting that PRP possesses anti-inflammatory and antimicrobial properties. To date the mechanism by which PRP achieves an antibacterial effect and promotes wound healing is not well understood.

## Methods

### Standard procedure and guidelines for systematic literature reviews

The literature review was performed based on the principles described in the Preferred Reporting Items for Systematic Reviews and Meta-Analyses guidelines, dated 2009.

Articles were screened for relevance using predefined inclusion/exclusion criteria. Search results were screened at 2 levels by 2 reviewers. Abstracts were reviewed for eligibility at the first level of screening. Relevant or possibly relevant articles were reviewed and assessed for inclusion at the second level of screening. Publications were evaluated for inclusion in the review regardless of whether the articles contained favorable or unfavorable findings concerning the PRP preparations. The following literature search terms were used: PRP, platelet-rich plasma, plasma concentrate, deep sternal wound infection, surgical wound infection, wound, infections, sternum, sternotomy, sternal reconstruction, antimicrobial, bacterial infections, microcidal, microbiota, anti-bacterial agents, with publication date from 2004/01/01 to 2019/08/31, and in English.

The following studies were excluded from data extraction: foreign language, full text not available, non-peer reviewed, case series/reports, not surgical or cutaneous wound healing, study with a mixed cohort, and where data specific to PRP could not be extracted. The review/systematic reviews and meta-analysis articles that were identified during the screening process were included in the background section but excluded from data evaluation.

## Information source and search strategy

The literature search was designed and performed independently by a library information specialist. Abstracts and articles were reviewed by 2 of the reviewers. The PubMed®, MEDLINE®, and EMBASE® databases were used to search the peer-reviewed medical literature.

EBSCO MEDLINE was systematically searched using the following Boolean Search terms: S1 TI (“platelet rich plasma” OR PRP OR “plasma concentrate”) OR AB (“platelet rich plasma” OR PRP OR “plasma concentrate”) OR MH “platelet rich plasma”; S2 TI (antimicrobial OR antibacterial OR microcidal OR anti-bacterial OR microbiota) OR AB (antimicrobial OR antibacterial OR microcidal OR anti-bacterial OR microbiota) OR MH (“bacterial infections” OR “anti-infective agents”); S3 TI (“deep sternal wound infection*” OR DSWI) OR AB (“deep sternal wound infection*” OR DSWI); S4 (MH “surgical wound infection” OR ((TI wound* OR AB wound*) AND (TI infect* OR AB infect*))) AND (MH (sternotomy OR “sternum”) OR TI (sternotom* OR “sternal reconstruction”) OR AB (sternotom* OR “sternal reconstruction”)); S5 S1 AND S2; S6 S1 AND (S3 OR S4); S7 S5 OR S6; S8 S7 AND LA English; S9 S8 AND DT 20040101-20190831.

There were some discrepancies between the planned search and the search executed. The modification was made as use of planned search terms led to limited articles. In addition, a manual search was carried out to retrieve other articles that had not been identified via initial search strategy and was uploaded to Distiller Systematic Review for screening purposes.

## Data management

Literature search results were uploaded to Distiller Systematic Review (DSR) software (Evidence Partners, Inc, Canada), an internet-based software program that facilitated collaboration among reviewers during the study selection process. The team developed and tested screening questions and forms for level 1 and 2 assessments based on the inclusion and exclusion criteria. Citation abstracts and full text articles were uploaded to DSR. Prior to the formal screening process, a calibration exercise was undertaken to pilot and refine the screening questions. Two authors independently screened the resulting articles based on the screening parameters set in DSR.

## Data collection and extraction

Using standardized forms developed in DSR, data were extracted independently from each eligible study to broadly address the research questions for this systematic literature review. The studies thereby selected were assessed for their appropriateness for inclusion and quality of method. The publication, type of study and sample size, study objective and outcome measure, PRP type used, types of bacteria targeted, type of activator or matrix used, cellular composition, and changes in outcome measures are shown for each study in Table 1 (bench experiments), Table 2 (preclinical studies), and Table 3 (clinical studies). Some of the studies that demonstrated antibacterial effect of PRP on dental/oral infections were excluded from the data extractions as the oral microenvironment is different from skin and/or surgical injections. Similarly, the review articles or meta-analysis reviews were utilized to summarize the clinical background for this systematic literature review but were not included in the data evaluation.

**Table 1:** Bench experiments summary

Author, year	Study type	Objective	PRP type versus control	Bacteria targeted	Results	Notes
Everts, 2006 [24]	In vitro (N = 10)	To compare properties of BC-PRP prepared using three different commercially available devices that are used to prepare platelet gel, specifically focused on the most important platelet growth factors and WBC-derived MPO	BC-PRP versus whole blood	Not studied	* Platelet and WBC yield was significantly increased by ECS and GPS * Nonsignificant increase in platelet and WBC count with AGF * Significant increase in TGF-β1 and PDGF level in PG samples after activation of the E-CS or GPS samples * No change in TGF-β1 and PDGF level in PG samples ECS-AGF samples * No significant differences between MPO concentrations in BC-PRP and whole blood * MPO concentration in the BC-PRP was significantly increased versus baseline (*P* < 0.001), indicating WBC activation before PG formation *No correlation of MPO release and cellular count	Healthy donors Statistics are unclear, could have randomized blood volunteers to devices
Bielecki, 2007 [38]	In vitro (N = 20)	To analyze antibacterial effect of PRP gel in vitro	PRP gel versus thrombin	* MRSA * MSSA * *Escherichia coli* * K. pneumoniae * E. Faecalis * P. aeruginosa	* PRP gel inhibited growth of *S. aureus* and *E. coli* * There was no activity against *K. pneumoniae*, *E. faecalis*, and *P. aeruginosa* * PRP gel seemed to induce in vitro growth of Aeruginosa, suggesting that it may cause an exacerbation of infections with this organism * No correlation between antimicrobial activity and concentration of platelets and leukocytes	Review paper
Moojen, 2008 [39]	In vitro (N = 6)	To investigate antimicrobial activity of PLG against *S. aureus* and the contribution of MPO present in leukocytes in this process	PLG versus PPP	*S. aureus*	* PLG-AT, PLG-BT, PRP inhibited the growth of *S. aureus* * There was release of MPO as early as 4 h in PLG-AT, PLG-BT, and PRP; MPO release was maximum at 8 h compared to PPP * No correlation between antimicrobial activity and MPO release and MPO activity	Unclear if blood from individual donors was pooled. No control for individual donor demographics or inclusion/exclusion criteria. Healthy donors may not translate to sick population
Tohidnezhad, 2012 [40]	In vitro (N = 14)	To investigate secretion of antimicrobial peptides by thrombocytes to elucidate the mechanism of thrombocyte anti-infective capabilities	PRP versus PRP	* *B. megaterium* * P. mirabilis * *Escherichia coli* * E. coli * K. pneumonia * E. faecalis	* PRP inhibited growth of *S. aureus*, *E. coli*, *B. megaterium* (*P* < 0.036), *P. aeruginosa* (*P* < 0.008), and *E. faecalis* (*P* < 0.001) and not *P. mirabalis* * hBD-3 concentration is significantly increased in PRP and PPP supernatant after activation and might act as first line defense	Young healthy donors may not translate. Pooled blood from the individual healthy donors. No control for individual demographics or inclusion/exclusion criteria
Tohidnezhad, 2011 [23]	In vitro (N = 24)	To investigate release of hBD-2 by platelets as a local antibacterial agent	PRP PRGF versus PPP	* *B. megaterium* * P. mirabilis * Escherichia coli * E. coli * K. pneumonia * E. faecalis	* PRP inhibited growth of *E. coli*, *B. megaterium*, *P. aeruginosa*, *E. faecalis*, and *P. mirabilis* * hBD-2 concentration is significantly increased in PRP compared to PPP and PRGF * Preincubation of *E. coli* and *P. mirabilis* significantly decreased antibacterial effect of PRP in these strains	Pooled blood from individual healthy donors expired platelets. No control for individual donor demographics or inclusion/exclusion criteria
Burnouf, 2013 [30]	In vitro (N = 2)	To compare the antimicrobial activity of four distinct plasma and platelet materials from 2 donors against 4 g-positive and 4 g-negative bacteria that can colonize wounds, and to elucidate which component in PG preparation can inhibit wound bacteria	PRP supernatant PG S/D-PL versus Inactivated PRP	* *P. aeruginosa* * E. coli * E. cloacae * K. pneumoniae * B. cereus * B. subtilis * S. aureus * S. epidermidis	* *E. coli* colony was strongly inhibited with native PRP, PPP, PG, and S/D-PL at 3 h * *P. aeruginosa* count was strongly reduced in native PRP, PG, and S/D-PL (4.62, 4.61, and 4.80 log, respectively) but much less in PG (1.10 log) * After 3 h, there was regrowth of *P. aeruginosa* in all PRP preparations * *K. pneumoniae* was strongly inhibited in native PRP, PPP, and S/D-PL (6.71, > 7.71, and 6.71 log, respectively) and less in PG (4.63 log) * *E. cloacae* growth was less affected by the plasma and platelet preparations (reduction close to or < 1 log) *Growth of the 4 g-negative bacteria was not inhibited when preparations were heat-treated to inactivate complement, suggesting the role of complement in bacterial inhibition *By contrast, a close to 100-fold inhibition of *S. aureus* was seen with native PRP, PPP, and S/D-PL (1.50, 2.10, and 1.80 log, respectively) but not with PG (0.23 log)	Healthy volunteers, no donor information, no inclusion/exclusion criteria
Li, 2013 [42]	Ex vivo (N = 50)	To investigate antibacterial property of L-PRP gel against MRSA in a rabbit model of osteomyelitis	L-PRP gel versus no treatment control	MRSA	*There was an increase in the concentrations of the 4 growth factors in the activated PRP versus whole blood and non-activated PRP *The highest VEGF levels in L-PRP gel supernatants were detected 1 h after activation (5.0-fold increase) *The highest PDGF-BB concentrations were observed in PRP supernatants 3 days after activation (3.4-fold increase), whereas TGF-β1 concentrations was highest at 1 day after activation *IGF-1 concentrations in supernatants from non-activated PRPs were higher than activated samples * Infection rate of control group was significantly higher than vancomycin (*P* = 0.035), L-PRP gel with vancomycin (*P* = 0.02), and L-PRP gel only (*P* = 0.088) * L-PRP gel could promote bone regeneration effectively only when infection was controlled	No increase in growth factors or cytokines in unactivated PRP versus whole blood except for IGF-1
Aktan, 2013 [25]	In vivo (N = 5)	To investigate equine platelets on bacterial growth, their ability to release products with antimicrobial properties or to influence the antimicrobial actions of neutrophils	L-PRP gel versus PRP and PPP, PBS	* *E. coli* * S. aureus	* PRP and PPP inhibited growth of *E. coli* and the effect was more prominent with activated PRP at 0.5 and 2 h * Phorbol myristate acetate stimulated platelets and caused increased superoxide production; significant platelet superoxide production was not observed in response to strong platelet stimuli such as thrombin and platelet activated factor * LPS and LTA activated platelets as measured with increased P-selectin expression * LPS and LTA had no effect on platelet superoxide production or heterotypic aggregate formation * Coincubation of activated platelets with neutrophils did not increase neutrophil superoxide production	Equine
Li, 2013 [41]	In vitro and ex vivo (N = 5)	To evaluate the antimicrobial and wound healing properties of PRP in spine infection rabbit model	PRP versus PBS	* MSSA * MSRA * Group A streptococcus * Neisseria gonorrhoeae	* PRP treatment has no significant antimicrobial effects against *E. coli* and Pseudomonas * PRP could significantly (80–100 fold reduction in CFUs at 200 IU/mL thrombin) inhibit the growth of MSSA, MRSA, Group A Streptococcus, and Neisseria gonorrhoeae within the first 2 h * The concentration of thrombin played a role in the antimicrobial properties of PRP; the higher the thrombin concentration (over the range of 20 to 200 IU/mL), the better the antimicrobial properties * PRP showed the capability to improve bone healing in the presence of a severe infection	Rabbit spine
Różalski, 2013 [31]	In vitro (N = 5)	To evaluate microbicidal activity of platelets and their products against *S. aureus* in suspension (planktonic) and sessile (biofilm) cultures	PRP (Expired: 1–3 post shelf life) versus Müller–Hinton agar	* *S. aureus*	* Microbicidal activity of “expired” platelets and their lysates has been shown as a significant reduction in the population of staphylococci in their planktonic cultures by 56–87% and a decrease in metabolic activity of biofilm formation by 7–38% * Antibacterial effect was enhanced after activation with ADP * Platelet lysates showed a synergistic effect with β-lactam antibiotic (oxacillin) and glycopeptide (vancomycin) but not with oxazolidinone (linezolid)	No control for donors, pooled many donors, expired platelets
Edelblute, 2014 [43]	In vitro and ex vivo (N = 3–7)	To quantify efficacy of human platelet gel against opportunistic bacterial wound pathogens *A. baumannii*, *P. aeruginosa*, and *S. aureus* on skin	PRP versus quiescent platelet pellet (minimally manipulated)	* A. baumannii * P. aeruginosa * S. aureus	* *A. baumannii* was significantly (*P* < 0.001) inactivated by both control and activated PG supernatants * *S. aureus* was significantly (*P* < 0.05) inactivated only by the thrombin- and PEF-activated supernatants * No significant inactivation was observed in the quiescent or CaCl_2_ enriched groups * *P. aeruginosa* was not inactivated in vitro; a low but significant inactivation level was observed ex vivo * PRP supernatants were quite effective at inactivating a model organism on skin in vivo	Well designed, expired platelets, multiple donors
Intravia, 2014 [28]	In vitro (N = 2)	To investigate antibacterial properties of 2 PRP: platelet concentration preparations (PRP-LP and PRP-HP)	* PRP-LP: lower WBCs and platelet concentration * PRP-HP: high platelet and WBC concentration vs * Blood + PBS (negative control) * Cefazolin (positive control)	* *S. aureus* * S. epidermidis * MRSA * *P. acnes*	* Both PRP-LP and PRP-HP showed a significant decrease (*P* < 0.05) in bacterial growth at 8 h compared to whole blood * There was no statistically significant difference between PRP-LP or PRP-HP and cefazolin at 24 h * The effect of PRP-LP and PRP-HP on *P. acnes* and MRSA was minimal and may not be clinically significant * Despite differences in platelet and WBC concentrations, no difference in antibacterial activity was seen between PRP-LP and PRP-HP preparations	Two healthy donors; low sample size, and healthy PRP may not translate
Frelinger, 2016 [9]	In vitro (N = 5)	To compare the ability of PEF, bovine thrombin, and TRAP to activate human PRP, release growth factors, and induce cell proliferation in vitro	PRP versus PRP treated with 0.9% sodium chloride, PPP	Not studied	* Both PEF and bovine thrombin reduced the average size of platelet-related (CD41-positive) particles, but the number of detectable CD41-positive particles after PEF activation was significantly higher than after bovine thrombin activation * Surface P-selectin was increased on particles following PEF activation but was increased to a greater extent by bovine thrombin and TRAP * PEF activation produced a higher number of procoagulant annexin V-positive particles. The total annexin V binding (a measure of phosphatidylserine expression) was similar after PEF and bovine thrombin activation * PEF activation of fresh PRP resulted in greater release of EGF than activation with bovine thrombin, whereas only TRAP activation resulted in significant release of angiostatin * Plasma containing the releasate from PEF-activated PRP induced significantly greater proliferation of an epithelial cell line than plasma recovered from vehicle-treated PRP	Healthy donors may not translate
Mariani, 2015 [14]	In vitro (N = 10)	To compare in vitro microbicidal activity of platelets and L-PRP to P-PRP and the contribution of leukocytes to microbicidal properties	PRP versus L-PRP cryo and P-PRP	* *E. coli* * S. Aureus * K. pneumoniae * P. aeruginosa * E. faecalis	* L-PRP, L-PRP cryo and P-PRP generally induced comparable bacterial growth inhibition for up to 4 h incubation * The concentrations of soluble factors considered (MIP-1α/CCL3, RANTES/CCL5, GRO-α/CXCL1, NAP-2/CXCL7, IL-8/CXCL8, SDF-1α/CXCL12 and IL-6) were strongly correlated to bacterial growth inhibition, mainly from the second hour of incubation * *E. coli* inhibition showed correlations with RANTES, GRO-α, and SDF-1α concentrations (*P* ≤ 0.05) * *S. aureus* inhibition correlated with the concentrations of all the molecules excluded IL-6 (*P* ≤ 0.05) * *K. pneumoniae*, *P. aeruginosa*, and *E. faecalis* inhibition correlated with the concentrations of all microbicidal molecules considered (*P* ≤ 0.05) * L-PRP and L-PRP cryo exhibited similar microbicidal activity	Healthy donors may not translate, correlation of bacterial growth to soluble factors
Lu, 2016 [36]	In vitro and ex vivo	To examine effects of a combined chitosan–gelatin sponge loaded with tannins and PRP (CSGT-PRP) on extent and rate of wound healing, antibacterial effects of the sponge, and stability of the wound dressing material	PDGF isolated from thrombocyte concentrate versus wound dressing and PRP, CSGT-PRP	* *E. coli* * S. aureus	* CSGT-PRP had good thermostability and mechanical properties as well as efficient water absorption and retention capacities * CSGT-PRP could effectively inhibit the growth of *E. coli* and *S. aureus* with low toxicity * CSGT-PRP healed wound quickly as observed macroscopically and by histological examinations	Healthy animals’ PRP may have different properties than those from infected animals
Bayer, 2016 [21]	In vitro and ex vivo	To determine if Chitosan composite hydrogel system is an effective medium for antibiotic delivery in wound infection caused by *S. aureus*	Vivostat PRF® (thrombocyte concentrate) versus 0.9% sodium chloride	None	* PRGF increases hBD-2 expression in concentration- and time-dependent manner * PRGF mediated hBD-2 expression was mediated through EGFR and interleukin receptor (IL-6) * hBD-2 indication through PDGF required activation of transcription factor activator protein (AP-1), but not through nuclear factor kappa ĸB * Vivostat PRF mediated wound healing is mediated through hBD-2 expression	Healthy donors, no demographic information on the donors
Nimal, 2016 [47]	In vitro	To develop a novel injectable hydrogel system for infectious wound treatment that can reduce the inflammatory phase (by inhibiting bacterial growth using tigecycline) and enhance the granulation phase (by addition of PRP) and simultaneously promote efficient wound healing	PRP from blood bank versus Tigecycline nanoparticles + chitosan hydrogel in PBS	*S. aureus*	* tg-ChPRP gel, and tg-ChNPs-ChPRP gel showed a significant zone of inhibition against *S. aureus* * ChPRP gel alone failed to demonstrate any antibacterial activity, and the incorporation of PRP in to the tg-Ch and tg-ChNPs-Ch gel did not enhance or inhibit antibacterial activity * ChPRP gel with the lowest concentration of tg-ChNPs (1 μg/mg) also showed a significant reduction in bacterial growth	No information on donor blood from blood bank; assume healthy non-infected donors
Knafl, 2017 [33]	In vitro (N = 5)	To evaluate release kinetics of amikacin, teicoplanin, or polyhexanide from a PRF layer	PRF versus Trypsin only and PRF only (negative control)	* MSSA * MRSA * *P. aeruginosa* * K. pneumoniae	* Teicoplanin and amikacin released from PRF showed antimicrobial in vitro effects for almost a week * Antimicrobial effect of polyhexanide could only be verified for the first 24 h	Small sample size and limited information on donor plasma
Bayer, 2018 [34]	In vitro	To demonstrate that PRGF induces antimicrobial peptides in primary keratinocytes and accelerates keratinocyte proliferation	Vivostat PRF® versus untreated using normal human epidermal keratinocytes cells	None	* PRGF stimulation caused a significant decrease in the Ki-67 gene expression in human primary keratinocytes, and EGFR is not essential to the PRGF-mediated reduction of Ki-67 gene expression in human primary keratinocytes * The interleukin-6 receptor (IL-6R) is not essential to the PRGF-mediated reduction of Ki-67 gene expression in human primary keratinocytes * IL-6 signaling is not involved in the PRGF-mediated reduction of the Ki-67gene expression in primary human keratinocytes	Healthy donors and no demographic information
Cetinkaya, 2018 [44]	Ex vivo (N = 72)	To investigate antibacterial activity and wound healing effectiveness of PRP in MRSA-contaminated superficial soft tissue wounds in Wistar Rats	PRP and vancomycin versus Sham, PRP, MRSA, MRSA + PRP, MRSA + vancomycin, MRSA + vancomycin + PRP	MRSA	* MRSA counts were lowest in MRSA + vancomycin + PRP groups * Inflammation scores of MRSA + PRP, MRSA + vancomycin, and MRSA + vancomycin + PRP groups were significantly lower than the MRSA group * The inflammation score was significantly lowest in MRSA + PPRP + vancomycin suggesting synergistic effect of vancomycin	No a priori sample size calculation
Cetinkaya, 2019 [45]	Ex vivo (N = 10)	To demonstrate in vitro antibacterial activity of PRP against MRSA and 3 more multi-drug resistant bacteria species that are important and hard-to-treat in wound infections	PRP gel versus PBS and PPP	* MRSA * Enterococcus spp * *K. pneumoniae* * P. aeruginosa	* PRP and PPP significantly suppressed bacterial growth of MRSA, *K. pneumoniae*, and *P. aeruginosa* as early as 1st, 2nd, 5th, and 10th hours of incubation (*P* < 0.05) compared to control * The antibacterial effect of PRP was more prominent compared to PPP * PRP and PPP showed limited activity against VRE	Healthy donors may not translate to clinical use
Cieślik-Bielecka, 2019 [46]	In vitro (N = 20)	To evaluate the antimicrobial effect of L-PRP against selected bacterial strains and assess potential correlation with leukocyte and platelet concentrations	L-PRP versus acellular plasma	* *S. aureus* * E. coli * Cryptococcus neoformans * Candida albicans	* L-PRP possesses an in vitro antimicrobial activity against MRSA, MSSA, *E. faecalis*, and *P. aeruginosa* * L-PRP did not exert any antimicrobial activity against *E. coli* (extended spectrum beta lactamase), *E. coli*, and *K. pneumoniae* * A relationship was observed among selected leukocyte subtypes (T and B lymphocytic NK cells, monocytes, and granulocytes with CD45) and L-PRP antimicrobial activity	Healthy donor plasma may not have properties of patients who have infection
Li, 2019 [29]	In vitro (N = 21)	To examine the potential mechanism underlying roles of PRP in treating diabetic foot ulcers	PRP and extract liquid of PRG versus PPP	* *S. aureus*	* PRG and EPG exhibited antibacterial effect against *S. aureus* * PRG and EPG protect HaCaT cells from bacterial damage and promote cell proliferation * Incubation of HaCaT cells with *S. aureus* decreased cell proliferation * The level of programmed cell death factor 4 and activity of NF-κB were increased in HaCaTcells with concomitant increased IL-6, TNF-α and decreased IL-10, TGF-β1 in cultured supernatant * EPG increased intracellular miRNA-21 while reducing PDCD4 expression and inhibiting NF-κB activity to suppress the inflammation in HaCaT cells * Both PRG and EPG had a significant reduction in bacterial count within 12 h (*P* < 0.01) compared to control	Used PRP from patients with infected foot ulcers
Ikono, 2018 [49]	In vitro	To assess use of chitosan-PRP nanoparticles to improve the viability of PRP and prolonged release of growth factors	Chitosan-PRP nanoparticles	*S. mutans*	* Chitosan PRP nanoparticles had strong antibacterial activity against *S. mutans* (90.63% inhibition), suggesting a novel mechanism to deliver PRP in wounds to promote healing	No data on donors

**Table 2:** Preclinical summary

Author, year	Study type	Objective	PRP type versus control	Bacteria targeted	Results
Nimal, 2016 [47]	Drosophila model	To develop a novel injectable hydrogel system for infectious wound treatment that can reduce the inflammatory phase (by inhibiting the bacterial growth using tigecycline) and enhance the granulation phase (by the addition of PRP) and simultaneously promote efficient wound healing	PRP from blood bank versus Tigecycline nanoparticles + chitosan hydrogel in PBS	*S. aureus*	* tg-ChPRP gel, and tg-ChNPs-ChPRP gel showed a significant zone of inhibition against *S. aureus*
					* ChPRP gel alone failed to demonstrate any antibacterial activity, and the incorporation of PRP into the tg-Ch and tg-ChNPs-Ch gel did not enhance or inhibit antibacterial activity
					* ChPRP gel with the lowest concentration of tg-ChNPs (1 μg/mg) showed a significant reduction in bacterial growth
Yassin, 2019 [48]	Rat model (N = 12)	To compare the efficacy of PRP wafers and PRP powder in terms of platelet count, antibacterial and healing effects in a rat model	PRP wafer and lyophilized PRP versus PRP-free wafer	*A. baumannii*	* PRP had antibacterial activity against *A. baumannii*
					* PRP wafer showed highest percent of wound size reduction on induced wounds in rat
					* PRP wafers achieved the shortest healing time followed by lyophilized PRP powder and finally PRP-free wafer
Shibata, 2018 [37]	Rabbit model (N = 16)	To evaluate the effectiveness of the controlled release of PRP from biodegradable gelatin hydrogel used to promote healing in a rabbit ischemic sternal model	PRP versus no treatment	Sternal healing, no particular bacteria flora studied	* PRP gel group showed a significantly higher proportion of fibrosis within the fracture area (an indicator of sternal healing) than the other groups
					* PRP significantly increased the mean intensity of osteocalcin, suggesting bone regeneration
Farghali, 2018 [50]	Canine (N = 6)	To compare healing and bacterial clearance of MRSA-infected wounds and wound tissue expression of TNF-α, and VEGFA and the concentration of malondialdhyde and gluthathione reductase in wound tissue over time	PRP versus clindamycin	MRSA	The PRP experimental group demonstrated superior healing by all measures: clinical examination/measurement, clinical examination, bacterial growth evaluation, biochemical assessment of oxidative stress, quantification of the expression of growth factor and cytokine genes, histopathological analysis, and immunohistochemical evaluation. PRP had a strong effect on MRSA; notably however, this effect was only observed when PRP was activated with CaCl_2_
Li, 2013 [41]	New Zealand rabbits (N = 50)	To compare MRSA-induced osteomyelitis treatment outcomes radiologically, microbiologically, and histologically	L-PRP gel versus no treatment, debridement and parenteral treatment with vancomycin (VAN) alone, L-PRP gel + VAN, L-PRP gel injection	MRSA	L-PRP gel + VAN group exhibited best therapeutic efficacy for bone healing and infection elimination. Best in vivo efficacy was for vancomycin, although PRP also demonstrated antibiotic efficacy

**Table 3:** Clinical trial summary

Author, year	Design, no. subjects	Indication	Objective	PRP type used	Control	Types of bacteria targeted	Results	Oxford level bias notes
Dorge, 2013 [5]	Randomized (N = 196) (underpowered and no a priori power and sample size estimate so no stopping criteria)	DSWI	To investigate whether topical application of autologous PRP reduced the incidence of DSWI in patients with high risk undergoing cardiac surgery with full sternotomy	PRP (N = 97)	Wound care (N = 99)	Not reported	* In PRP group 6 (6.2%) patients had DSWI versus 3 (3.0%) patients in control group	2b
Serraino, 2015 [32]	Retrospective (N = 1093)	DSWI	To evaluate whether PRP applied inside the sternotomy wound would reduce the effect of sternal wound infections, both superficial and deep	PRP	Median sternotomy but without PRP application	Not reported	* Occurrence of DSWI was significantly higher in control than PRP group (10/671, 1.5% versus 1/422, 0.2%, *P* = 0.043)	2b
							* Superficial sternal wound infections (SSWIs) were significantly higher in control than PRP group (19/671, 2.8% versus 2/422, 0.5%, *P* = 0.006)	
							* PRP can significantly reduce occurrence of DSWI and SSWI in cardiac surgery	
Hamman, 2014 [3]	Retrospective (N = 1866)	Severe DSWI	To evaluate impact of vancomycin, calcium-thrombin, and PRP in practice on incidence of severe DSWIs in a single surgeon’s patient population	PRP (N = 548)	Historical control (N = 1318) received routine antibiotic prophylaxis	Not reported	* Overall, 11 patients (0.59%) developed severe DSWIs (categories 5 and 6) in control group	2c
							* No severe incidence of DSWI in the intervention group (548 patients)	
Patel, 2016 [4]	Prospective (N = 2000)	DSWI	To analyze addition of PRP using a rapid point of care bedside system to standard wound care in all patients undergoing sternotomy for cardiac surgical procedures	Autologous PRP (N = 1000)	Standard of care sternal closure including preoperative antibiotics (N = 1000)	Not reported	* PRP reduced incidence of DSWI from 2.0 to 0.6%, SWI from 8.0 to 2.0%, and readmission rate from 4.0 to 0.8%	2b
							* PRP reduced costs associated with development of deep and superficial wound complications from $1,256,960 to $593,791	
Wozniak, 2016 [51]	Prospective (N = 34)	Leg ulcers	To perform qualitative analysis of microbial flora in venous leg ulcers treated with PRP	PRP	Pretreatment microbial flora	* *P. aeruginosa*	* PRP given as intradermal injection and after PRP therapy, a significant healing improvement was shown in 21 subjects (61.8%), as assessed by decrease in wound size	4; underpowered
						* S. aureus	* There was no improvement in 13 subjects (38.2%)	
						* E. faecalis	* *S. aureus* and *P. aeruginosa* were most commonly identified bacteria	
						* B. fragilis	* 83 different microbial flora were identified from wound	
						* 83 microbial flora identified from wound	* After PRP therapy, 110 bacterial isolates were obtained from samples collected after a single, local application of PRP	
							* The mean number of bacterial species isolated per subject was 2.44 ± 0.22 before and 3.24 ± 0.29 after PRP	
Englert, 2005 [53]	Prospective, randomized (N = 34)	Cardiovascular surgery	To examine effects of autologous platelet gel on postoperative sternal wound healing by subjective reports of chest and leg pain, amount of measurable bruising, and platelet indices pre- and postoperatively	PRP gel	Standard of care		* There was a significant decrease in chest and leg pain with PRP treatment	4
							* PRP decreased bruising but was not statistically significant compared to control	
Tran, 2014 [54]	Prospective, single arm (N = 6)	Diabetic foot ulcer	To examine effects of autologous platelet gel on postoperative sternal wound healing by subjective reports of chest and leg pain, amount of measurable bruising, and platelet indices pre- and postoperatively	PRP gel	Not reported		* PRP and PPP application caused increased wound healing (100%) by week 7	4
Vang, 2017 [55]	Prospective, randomized (N = 38)	Surgical wound healing	To examine whether autologous platelet gel to sternum and saphenous vein harvest site was beneficial to patients undergoing coronary artery revascularization in terms of pain, blood loss, discoloration, and surgical site infection	PRP gel	Standard wound care without PRP		* 8 patients discontinued the study due to complication with coronary artery bypass graft surgery	4
							* There was no incidence of deep or superficial sternal infections	
							* One patient from treatment and control group experienced a leg infection at the saphenous vein harvesting site after hospital discharge	
							* Patients reported less postoperative pain in PRP group versus control group	

All articles that have reported preclinical (in vitro and ex vivo) and clinical studies are included. In vitro study was defined as the technique that is performed in a controlled environment outside of a living organism without being implanted again into the living body or organism. Ex vitro study was defined as the technique that is performed in a controlled environment inside of a living organism. Clinical studies are conducted in humans.

## Data synthesis

There was heterogenicity among articles selected for the data evaluation. Due to the lack of homogeneity among the resulting studies, a meta-analysis could not be performed. Therefore, all studies that met the inclusion criteria have been presented in a narrative synthesis, which represents a wide variety of studies where conclusions are based on reason or argument.

## Study selection, risk of bias, and quality assessment

Two authors independently screened the resulting articles for their methodologies and appropriateness for inclusion and exclusion. In cases of discrepancies, consensus was reached by discussion between the reviewers, with a third reviewer serving as arbiter if an agreement could not be reached.

The Cochrane Risk of Bias in Non-Randomized Studies of Interventions (ROBINS-1) was used to assess bias in non-randomized clinical trials. The Oxford Center for Evidence Based Medicine-Levels of Evidence for a therapeutic was used to assess the overall quality of the clinical studies (Tables 4, 5 and 6) [26]. A modified Cochrane (clinical trial) risk of bias instrument called the Systematic Review Centre for Laboratory Animal Experimentation (SYRCLE) risk-of-bias tool was utilized for preclinical studies [27]. Studies were assigned a “Yes” for each applicable criterion they met, “No” for each they did not, and unclear” or “not indicated” for the studies containing insufficient information (Table 7). A “No” indicates more bias and a “Yes” indicates less bias.

**Table 4:** Oxford levels of evidence [26]

Level	Therapy/prevention/etiology/harm
1a	Systematic review (SR) (with homogeneity*) of RCTs
1b	Individual randomized controlled trial (RCT) (with narrow confidence interval”)
1c	All or none§
2a	SR (with homogeneity*) of cohort studies
2b	Individual cohort study (including low quality RCT; eg, < 80% follow-up)
2c	“Outcomes” Research; Ecological studies
3a	SR (with homogeneity*) of case–control studies
3b	Individual Case–Control Study
4	Case-series (and poor-quality cohort and case–control studies§§)
5	Expert opinion without explicit critical appraisal, or based on physiology, bench research or “first principles”

See Table 6 for definition of symbols

**Table 5:** Oxford grades [26]

A	Consistent level 1 studies
B	Consistent level 2 or 3 studies *or* extrapolations from level 1 studies
C	Level 4 studies *or* extrapolations from level 2 or 3 studies
D	Level 5 evidence *or* troublingly inconsistent or inconclusive studies of any level

Users can add a minus-sign “ − ” to denote the level that fails to provide a conclusive answer because: *either* a single result with a wide confidence interval *or* a systematic review with troublesome heterogeneity such evidence is inconclusive, and therefore can only generate graded recommendations

**Table 6:** Oxford definitions [26]

*	By homogeneity we mean a systematic review that is free of worrisome variations (heterogeneity) in the directions and degrees of results between individual studies. Not all systematic reviews with statistically significant heterogeneity need be worrisome, and not all worrisome heterogeneity need be statistically significant. As noted above, studies displaying worrisome heterogeneity should be tagged with a “-” at the end of their designated level
“	Clinical Decision Rule. (These are algorithms or scoring systems that lead to a prognostic estimation or a diagnostic category)
“¡	See note above for advice on how to understand, rate and use trials or other studies with wide confidence intervals
§	Met when all patients died before the Rx became available, but some now survive on it; or when some patients died before the Rx became available, but none now die on it
§§	By poor quality cohort study we mean one that failed to clearly define comparison groups and/or failed to measure exposures and outcomes in the same (preferably blinded), objective way in both exposed and non-exposed individuals and/or failed to identify or appropriately control known confounders and/or failed to carry out a sufficiently long and complete follow-up of patients. By poor quality case–control study we mean one that failed to clearly define comparison groups and/or failed to measure exposures and outcomes in the same (preferably blinded), objective way in both cases and controls and/or failed to identify or appropriately control known confounders
§§§	Split-sample validation is achieved by collecting all the information in a single tranche, then artificially dividing this into “derivation” and “validation” samples
” “	An “Absolute SpPin” is a diagnostic finding whose Specificity is so high that a Positive result rules-in the diagnosis. An “Absolute SnNout” is a diagnostic finding whose Sensitivity is so high that a Negative result rules-out the diagnosis
“¡”¡	Good, better, bad and worse refer to the comparisons between treatments in terms of their clinical risks and benefits
”” “	Good reference standards are independent of the test and applied blindly or objectively to applied to all patients. Poor reference standards are haphazardly applied, but still independent of the test. Use of a non-independent reference standard (where the ‘test’ is included in the ‘reference’, or where the ‘testing’ affects the ‘reference’) implies a level 4 study
””” “	Better-value treatments are clearly as good but cheaper, or better at the same or reduced cost. Worse-value treatments are as good and more expensive, or worse and the equally or more expensive
**	Validating studies test the quality of a specific diagnostic test, based on prior evidence. An exploratory study collects information and trawls the data (eg, using a regression analysis) to find which factors are ‘significant’
***	By poor quality prognostic cohort study we mean one in which sampling was biased in favor of patients who already had the target outcome, or the measurement of outcomes was accomplished in < 80% of study patients, or outcomes were determined in an unblinded, non-objective way, or there was no correction for confounding factors
****	Good follow-up in a differential diagnosis study is > 80%, with adequate time for alternative diagnoses to emerge (for example 1–6 months acute, 1–5 years chronic)

**Table 7:** Systematic Review Centre for Laboratory Animal Experimentation (SYRCLE) evaluation of bias for preclinical studies

Author year	Selection bias				Performance bias		Detection bias		Attrition bias: complete outcome data?	Reporting bias: non-selective outcome reporting?	No other sources of bias?
	Randomization?	Random sequence generation?	Baseline characteristics equal?	Allocation concealment?	Random housing?	Blinding?	Random selection outcome assessment?	Blinding?			
Yassin, 2019 [48]	No	No	No	No	No	No	No	No	Yes	Yes	Yes
Farghali, 2018 [50]	No	No	Yes	No	No	No	No	No	Yes	Yes	Yes
Shibata, 2018 [37]	Yes	No	Yes	No	No	No	No	No	Yes	Yes	Yes
Nimal, 2016 [47]	No	No	No	No	No	No	No	No	Yes	Yes	Yes
Cetinkaya, 2018 [45]	Yes	No	Yes	No	No	No	No	No	Yes	Yes	Yes

“Yes” indicates reduced bias and “No” indicates increased bias

## Literature search results

## Study selection

The data collection process was systematic and pre-specified. An initial search of PubMed®, MEDLINE®, and EMBASE® yielded 526 total articles for review, and 7 articles were subsequently added after a manual hand search of the articles. After initial screening, 195 papers were excluded for being in a language other than English, abstract or full text unavailability, non-peer reviewed articles, case series/report, not deep sternal wound infection (DSWI) or wound healing indication. The second level of screening excluded 28 articles for different reasons (mentioned above), such as full articles in another language, not pertaining to wound healing, pertaining to wound healing but not with PRP utilization, or PRP used in dental/oral conditions (due to different microenvironment as compared to surgical treatment). Six review articles were excluded from data extraction but were included for introduction section. Thus, at the end of the screening process, 34 articles were available for systematic review and narrative analysis that used PRP in antibacterial effect and wound healing in general and DSWI.

**Fig. 1:**
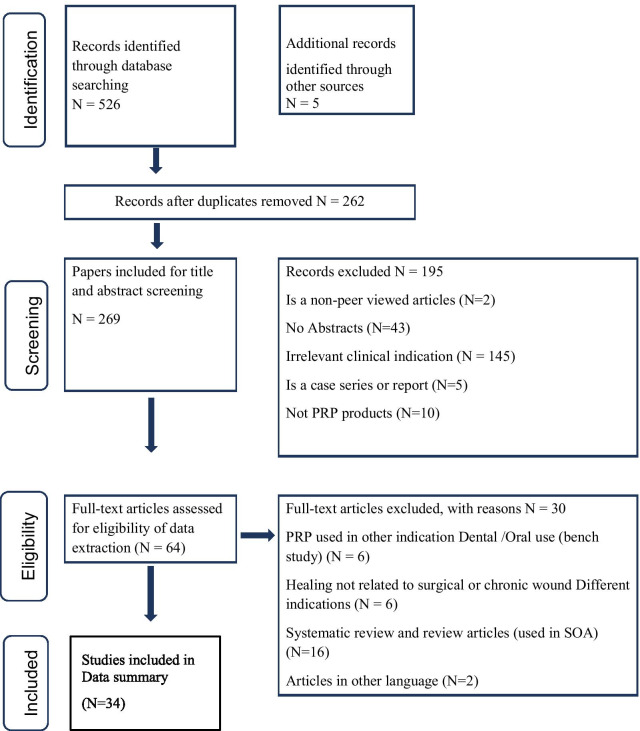
Flow chart summarizing the study selection procedures

A flow chart summarizing the search results, including the number of articles excluded at each stage of the review and the final number of included articles, is shown in Fig. 1.

## Variable methods of PRP separation

Different authors utilized various methods to process the whole blood collected from healthy or ill donors or animals to prepare PRP or buffy coat PRP (BC-PRP). PRP preparation involved 2 sequential centrifugation steps: separation and concentration. In the separation phase, initial centrifugation separates RBCs and the second spin concentrates cellular pellets consisting of leukocytes (neutrophils, lymphocytes, and monocytes), and platelets that are re-suspended in a small volume of the remaining PPP. For some studies, the following commercially available devices were used in preparation of PRP: Harvest SmartPReP2 System (Terumo Blood and Cell Technologies) [3], The Gravitational Platelet Sequestration System™ (GPS) Platelet Concentrate System (Biomet Biologics) [24, 28], Angel Whole Blood Processing System™ (Sorin Group, Italy), and Magellan® Autologous Platelet Separator System [4]. Other commercially available devices that were utilized to prepare BC-PRP were Electa Cell-Separator™ (ECS) (Sorin Group, Mirandola) and the Autologous Growth Factor filter™ (AGF) (Interpore Cross™, Irvine, CA). Other studies utilized a fully automatic blood separator [29], Apheresis machine MCS + (Haemonetics Corps) [30]. One study used frozen PRP produced from liquid-preserved platelet concentrates obtained by plateletpheresis [23] and 2 studies used expired platelet concentrates [31, 32]. Some studies utilized manual processing of the whole blood after collection. In general, there was variability in procedure times (3–20 min), centrifugation speed (305–3800 g), temperatures (20–26 °C), and cycles of centrifugation (single or double cycles). Some studies have utilized platelet-rich fibrin (PRF) [23, 33, 34], which consists of a fibrin matrix, platelet-derived cytokines, growth factors, and entrapped leukocytes devoid of erythrocytes and is prepared without addition of anticoagulants [33].

Overall, there was variability in methods to concentrate platelets and leukocytes. The concentration of platelets and leukocytes in the processed products ranged from ~ 0.5 × 10^6^ cells/mL to 9000 × 10^6^ cells/mL, and ~ 1.1 × 10^6^ cells/mL to 1350 × 10^6^ cells/mL, respectively. One group suggested that the variability in processing methods might prematurely activate platelets, altering the regenerative capacities of the final PRP-based product [35]. It is possible that a higher cell count of platelets or leukocytes does not always ensure a high concentration of growth factors in the PRP-based final product. Hence, the methodological variances in preparing PRP make it challenging to relate the results from different studies.

Activation of PRP is necessary to form a fibrin matrix for platelet attachment and adhesion. Activation of PRP is also crucial for the bioactivation of PRG that results in degranulation, the release of the substances and growth factors that contribute to the wound healing cascade, and the antibacterial effects of PRP. There was considerable variability among the methods utilized to activate PRP to form PRG, with authors using different activators and/or mechanical methods. Autologous thrombin, bovine thrombin, calcium chloride (0.5–10%), calcium gluconate, and calcium citrate were the most commonly used activators. The amount of activator added, and the time of activation varied among studies. Some studies used a single activator (calcium chloride or thrombin alone) while others utilized multiple activators in various proportions (combination of thrombin and calcium chloride). Mechanical methods of activation such as application of electric field pulse [9] or freeze/thaw cycle were also used [31]. One study utilized supernatant of the PRG formation but not the PRG itself [29].

PRP has been used in conjunction with antibiotics and in various physical forms, introducing additional substantial heterogeneity into the preclinical and clinical trials. PRP was used in combination with vancomycin [3], amikaycin/teicoplanin [33], and polyhexanide [33] and as a porous scaffold of Chitosan [36], a gelatin hydrogel [37], a PRP wafer, and a lyophilized PRP powder. These methodological differences in delivering PRP make it challenging to define a dose and/or administration protocol to validate.

## Bench experiments

Evert et al. compared several properties of BC-PRP from healthy donors (N = 10) using 3 commercial systems: ECS, The GPS, and the third system combined ECS prepared BC-PRP and processed through the AGF [24]. The study showed that the level of growth factors such as PDGF and TGF-β1 were present in high levels in PRG only after activation of the ECS or GPS PRP samples (*P* < 0.001). However, AGF-prepared PRP samples showed higher levels of PDGF and TGF-β1 before the activation, which might be the result of passing the platelets and leukocytes through the fibers of the AGF filtration system. Similarly, platelet recovery was lowest in AGF. The authors suggested that this could be due to the recurring passage of the platelets and leukocytes through fibers of the AGF filtration system used to concentrate the BC-PRP. There was an increase in WBC yield and the pattern of increase was similar between ECS and GPS (*P* < 0.001); however, there was a non-significant increase in WBC yield with ECS-AGF. According to their results, the total number of WBCs, neutrophils, monocytes, and lymphocytes significantly decreased in platelet gel (PG). To correlate the WBC after thrombin/calcium chloride activation, myeloperoxidase (MPO) concentration was measured. There was no significant difference in MPO concentration between PG (ECS and GPS), indicating that thrombin addition does not cause degranulation of WBCs. However, the level of MPO was higher in both BC-PRP and PRG processed using AGF, suggesting that leukocytes might prematurely be activated due to processing using AGF.

Bielecki et al. studied the antibacterial effect of PRP (N = 20) and PG in different bacterial strains including: methicillin-resistant and sensitive *S. aureus* (MRSA and MSSA), *E. coli* (extended spectrum beta lactamase), *E. coli*, *Klebsiella* (*K*) *pneumoniae*, *Enterococcus faecalis* (*E. faecalis*), and *P. aeruginosa* [38]. The authors collected blood from healthy volunteers to prepare PRP using the GPS. There was 7.6-fold increase in the mean platelet number and a 7.9-fold increase in leukocyte yield in PRP preparation compared to whole blood. PG was active against the growth of *S. aureus* and *E. coli* in comparison with thrombin alone; however, PG showed no effect against *K. pneumoniae*, *E. faecalis*, and *P. aeruginosa* strains. In fact, the authors reported that *P. aeruginosa* regrowth was observed after addition of PG, suggesting that PG might exacerbate infections related to this organism. There was no antibacterial effect of thrombin alone in any of the bacterial strains tested. In this study, the investigators were unable to report a direct correlation between the degree of antimicrobial activity of PRP and the platelet and leukocyte count in the whole blood or PRP.

Moojen et al. studied the antimicrobial activity of platelet leukocyte gel (PLG) against *S. aureus* and the contribution of MPO. PRP and PPP were prepared from whole blood from 6 healthy donors [39]. To explore the effect of different types of thrombin, PLG was prepared by mixing PRP with autologous thrombin (PLG-AT) or bovine thrombin (PLG-BT), while phosphate-buffered saline (PBS) served as control. The result showed a rapid decrease in bacterial count (log CFU/mL) for PGL-AT, PLG-BT, and PPP. The maximum antibacterial effect for PLG-AT and PLG-BT was observed as early as 4 and 8 h, and the effect was largest at 12 h for PRP alone and PPP alone, suggesting activation positively affected the efficacy of PRP. The antibacterial effect of PLG-AT was significantly larger compared to PRP alone (*P* < 0.004) or PPP alone (*P* < 0.001), however, and similar to that of PLG-BT (*P* < 0.93). The study showed that at 24 h, bacterial growth reached the stationary phase for all groups. To investigate the role of MPO in antibacterial effect of PRP, the authors measured the release and activity of MPO using the Muller Hinton broth culture medium. The authors reported gradual release of MPO as early as 4 h in PLG-AT, PLG-BT, and PRP alone, and MPO release was maximum at 8 h compared to PPP alone. MPO activity was comparable among PRP preparations (PLG-AT, PRP only, and PPP only). The authors reported no correlation between MPO release, MPO activity, and antibacterial effect of PRP preparations. The authors concluded that PRP is safe to use in patients and has antibacterial activity that might be effective to prevent postoperative infection.

Tohidnezhad et al. evaluated the antimicrobial effect of PRP against less common gram-negative microbes including *E. coli*, *Bacillus* (*B*) *megaterium*, *P. aeruginosa*, *E. faecalis* and *Proteus mirabilis* (*P. mirabilis*) that frequently colonize wounds after orthopedic trauma surgery (N = 3) [23]. In the study PRP was prepared using the liquid-preserved thrombocyte concentrate obtained by plateletpheresis and was activated by bovine thrombin and calcium chloride. Human keratinocytes served as internal positive controls and whole blood and PPP were also used as controls. PRP effectively inhibited the growth of *E. coli* (*P* < 0.015), *B. megaterium* (*P* < 0.036), *P. aeruginosa* (*P* < 0.008), and *E. faecalis* (*P* < 0.001) compared to whole blood except for *P. mirabalis*. To investigate the role of human beta defensin-3 (hBD-3) in PRP-mediated antibacterial effect, hBD-3 concentration was measured in PRP and PPP supernatant. The results demonstrated that the hBD-3 concentration was significantly higher in PRP (6146.3 ± 944.4 pg/mL) compared to PPP (2845.4 ± 1781.2 pg/mL) (*P* < 0.001) supernatant. The authors hypothesized that antibacterial effect could be mediated via the secretion of hBD-3 as a first-line defense in contaminated wounds and in elective application of PRP.

In another study Tohidnezhad and coworkers evaluated the release of human beta defensin-2 (hBD-2) as a local antimicrobial substance following PRP treatment (N = 8) [40]. The result showed that PRG significantly inhibited the growth of *E. coli*, *B. megaterium*, *P. aeruginosa*, *E. faecalis*, and *P. mirabilis* as indicated by the zone of inhibition of 6.44 ± 1.30, 7.84 ± 2.08, 5.00 ± 0.71, 3.73 ± 0.41, 6.50 ± 0.05, respectively, compared to the negative control. To investigate the role of hBD-2 in the antibacterial effect of PRP, the authors measured the level of hBD-2 in the PRP supernatant following activation with thrombin. hBD-2 concentration significantly increased in activated PRP supernatant (471 pg/10^9^ platelets) compared to PPP (221 pg/10^9^ platelets) and in platelet-released growth factors (PRGF) (188 pg/10^9^ platelets) (*P* < 0.0001). To confirm the role of hDB-2, bacteria were preincubated with anti hBD-2 antibodies. There was a significant decrease in antibacterial activity of PRP against *E. coli* and *P. mirabilis* (*P* < 0.05). This suggests that other antimicrobial peptides might participate in antibacterial effect in combating these strains.

Burnouf et al. compared the antibacterial effect of 4 distinct plasma and platelet preparations: PRP, activated PG supernatant, solvent/detergent-treated and virally inactivated platelet concentrate (S/D-PL) against the following gram-positive and gram-negative bacterial strains: *S. aureus*, *E. coli*, *K. pneumoniae*, *E. faecalis* and *P. aeruginosa* [30]. The authors reported that PPP, PRP, and S/D-PL have similar total protein, fibrinogen, immunoglobulins, and albumins, while PG has depleted fibrinogen and coagulation factors. To understand the role of plasma complement, the authors heat-inactivated PRP, PPP, PG, and S/D-PL samples (30 min at 56 °C) to inactivate the complement and treated bacterial strains and compared to native complement levels. Non-activated PRP was used as control of the starting material and also to determine if platelets affect bacterial growth. Nutrition broth and PBS were also used as controls where applicable. The platelet counts, WBC, and RBC counts were higher in PRP compared to PPP and there were no detectable blood cells in PRG S/D-PL and complement-inactivated products. The results suggested that platelet preparations exhibited antibacterial effect as early as 3 h. There was no detectable *E. coli* in native PRP, PPP, PG, and S/D-PL at 3, 24, and 48 h corresponding to greater than 7.5-log reduction compared to control. In contrast, a close to 100-fold inhibition of *S. aureus* was seen with native PRP, PPP, and S/D-PL (1.50, 2.10, and 1.80 log, respectively) but not with PRG (0.23 log). Similarly, *P. aeruginosa* and *K. pneumonia* were strongly inhibited by PRP, PPP, and S/D-PL but less by PRG, suggesting that plasma components might play a stronger role in bactericidal effect of platelet preparation, which might not be strongly correlated to platelets and WBC counts. This was further confirmed by treating these bacterial strains with heat-inactivated platelet preparations, where heat-inactivated PRP did not inhibit the growth of *Enterobacter cloacae* (*E. cloacae*), *B. cereus*, *B. subtilis*, *S. aureus*, and *S. epidermis*. Finally, the authors concluded heat-sensitive peptides or proteins most likely belonging to the complement systems play a major role in the inhibition of *E. coli*, *P. aeruginosa*, *S. aureus*, and *K. pneumoniae*.

Li et al. investigated the efficacy of leukocyte- and platelet-rich plasma (L-PRP) gel against MRSA using a rabbit model of osteomyelitis (N = 40) [41]. The whole blood collected from 10 rabbits was centrifuged to prepare PRP and L-PRP, which resulted in a 7.2-fold and 5.0-fold increase in platelet count and leukocyte count compared to whole blood. The osteomyelitis animal model was created by injecting MRSA and animals were randomized to 5 groups: control (no treatment) (N = 10), vancomycin (N = 10), L-PRP (N = 10), vancomycin + L-PRP (N = 10), and L-PRP gel alone (N = 10). There was a significant increase in 4 growth factors (VEGF, PDGF-BB, IGF-1, and TGF-β1) in L-PRP gel, suggesting that L-PRP thrombin activation of PRP releases growth factors. There was a threefold increase in VEGF, 3.4-fold increase in PDGF-BB, no change in IGF-1, and 4.4-fold increase in TGF-β1 in L-PRP gel compared to whole blood. The authors reported that there was a significant change in infection in rabbits receiving vancomycin (*P* < 0.02), L-PRP gel only (*P* = 0.088), and the lowest infection was observed in the L-PRP gel + vancomycin group, suggesting the synergistic effect of vancomycin and PRP. In the study, L-PRP injection alone also exhibited infection prevention although the effect was more prominent in the vancomycin only group. Similarly, the authors also reported that the bone window eventually healed, indicating that L-PRP gel could promote bone regeneration effectively only when infection was controlled. The authors concluded that L-PRP gel with high platelet and WBC is not only effective in promoting soft tissue and wound healing but also in interaction with bacterial contaminants.

Aktan et al. evaluated the effects of equine platelets on bacterial growth of *E. coli* and *S. aureus* and their ability to release products with anti-microbial properties [25]. Both PRP and PPP significantly inhibited the growth of *E. coli* following activation with thrombin. To determine if *E. coli*-derived lipopolysaccharide (LPS) and *S. aureus*-derived lipoteichoic acid (LTA) activate platelets, the authors evaluated expression of P-selectin. The result showed that LPS and LTA activate platelets as shown by increased P-selectin expression; however, LPS and LTA failed to increase platelet superoxide production or heterotypic aggregate formation following activation with thrombin. The study also reported that co-incubation of activated platelets with neutrophils did not increase neutrophil superoxide production, but platelets enhanced superoxide anion release from equine neutrophils, which was demonstrated by measuring phagocytosing opsonized zymosan. The authors concluded that equine platelets are capable of releasing ROS that could assist in bacterial killing.

Li et al. evaluated the antibacterial effect and wound healing property of PRP in bacterial strains (MRSA, MSSA, *E. coli*, group A streptococcus, Pseudomonas, *Neisseria gonorrhoeae*) isolated using a spine infection model. To understand the role of thrombin in PRP activation, the authors tested different concentrations of bovine thrombin (20, 100, and 200 IU/mL) [42]. The results demonstrated that there was a correlation between thrombin concentration and PRP activation as fewer granules were observed in platelets with increased concentration of thrombin. PRP following activation decreased in colony formation units (CFU) of MRSA, MSSA, group A streptococcus, and *Neisseria gonorrhoeae* in the first 2 h compared to control. PRP did not show any significant effect in *P. aureus* and *E. coli* whereas PPP did not show any effect in any of these bacterial strains. Similarly, in animal models the surgical site with bacterial challenge showed elevated bumps but the bumps were relatively smaller in size in the PRP-treated group, illustrating improved bone healing. The authors concluded that PRP has antimicrobial properties and that its antimicrobial properties are bacterial strain specific.

Edelblute et al. studied the efficacy of human platelet gel supernatant against opportunistic wound pathogens *Acinetobacter* (*A*) *baumannii*, *P. aeruginosa*, and *S. aureus* on skin [43]. The authors used the supernatant from the expired platelets and compared the efficacy of different activators (10% calcium chloride or bovine thrombin or platelet electric field [PEF]). Minimally manipulated quiescent platelet pellets were used as control. The study showed that human platelet gel supernatants were highly bactericidal against *A. baumannii* (*P* < 0.001) and moderately significant decrease in *S. aureus* (*P* < 0.051) but growth of *P. aeruginosa* was not affected except for platelet gel supernatant activated by PEF. A low yet significant inactivation level was observed in an ex vivo model. The authors also reported that the supernatants collected from activated PRP were effective at inhibiting the growth of bacteria on skin in vivo with respect to control (*P* < 0.05). The authors suggested that the minimal difference in antibacterial activity from control and treatment gels might support the idea that platelets were preactivated during the collection and storage.

Intravia et al. investigated antibacterial properties of two different PRP preparations: with low (PRP-LP) and high platelets (PRP-HP) (N = 2) [28]. Whole blood was obtained from 2 donors and processed to prepare PRP as follows: a single spin process yielding lower WBCs and platelet concentration (PRP-LP) (autologous conditioned plasma) and high platelet yield and WBC concentration preparation (PRP-HP) (GPS II, Biomet). The results showed that with PRP-LP and PRP-HP there was a ~ 3- to sixfold increase in platelets and ~ 0.11- to 2.2-fold increase in WBCs. Both PRP-LP and PRP-HP showed significant decreases in bacterial growth of *S. aureus*, *S. epidermis*, MRSA, and *Propionibacterium acnes* (*P. acnes*) (*P* < 0.05) at 8 h. PRP preparation was equally efficacious in inhibiting the growth of bacterial strains tested as cefazolin antibiotics. The study concluded that despite differences in platelet and WBC concentration between PRP-LP and PRP-HP, there was no substantial difference in antibacterial activity of these 2 PRP preparations, suggesting that the numbers of platelets and leukocytes do not directly affect antibacterial activity.

Frelinger et al. estimated the ability of PEF, bovine thrombin, and thrombin receptor-activating peptide (TRAP) to activate human PRP and compared the release of procoagulants markers, growth factors and the capacity of PRP on cell proliferation (N = 5) [9]. PRP was prepared using Harvest SmartPReP 2 system following manufacturer’s instructions. PRP treated with 0.9% NaCl was used as vehicle control and PRP treated with Triton X-100 was used to assess total growth factor levels. The results showed that P-selectin and CD41-positive particles were significantly increased by PRP-activated different methods mentioned previously. Similarly, the authors also reported that the release of proangiogenic growth factors (PDGF and EGF) and anti-angiogenic growth factor (PF4) was significantly higher in activated PRP (*P* < 0.05) compared to control. The study also found that factors released from PFE-activated PRP, but not bovine thrombin-activated or TRAP-activated PRP, significantly increased cell proliferation compared to control (*P* < 0.05), as indicated by increased annexin V-positive particles. A significant correlation was reported between the level of PDGF present in the lysate and cell proliferation. The authors concluded that PEF may be a superior alternative to the current standard of bovine thrombin for activation of therapeutic PRP.

Mariani et al. compared in vitro antibacterial activity of L-PRP and pure PRP (P-PRP) and studied the contribution of leukocytes to microbial properties in the following bacterial strains: *E. coli*, *S. aureus*, *K. pneumoniae*, *P. aeruginosa*, and *E. faecalis* (N = 10) [14]. The whole blood was collected and processed manually by single centrifugation to obtain P-PRP and by double centrifugation to obtain L-PRP. To reflect the real-world scenario where hemoderivatives could be used for a patient’s treatment after collection and storage, the authors utilized the cryopreserved fraction (L-PRP cryo) of PRP as well. L-PRP, P-PPP, and L-PRP cryo exhibited antibacterial effect for up to 4 h, depending on the bacterium. The growth inhibition ranged between 1 and 4 Log (corresponding to inhibitions from 10 to 10.000 CFU/mL); however, there was no growth inhibition with the PRP preparation after 18 h. The authors also evaluated the release of microcidal proteins. The result showed that there was a strong correlation between the microcidal protein release (such as CCL-3/MIP-1α, CCL-5/RANTES, CXCL/Gro-α, CXCL-8/IL-8, CXCL-7/NAP-2, CXCL/SDF-1α, and IL-6) and bacterial inhibition as early as 2 h. *E. coli* inhibition showed correlation with RANES, Gro-α, and SDF-1 α concentration. The inhibition of *S. aureus* growth showed significant correlation with all microcidal proteins released (*P* < 0.01 to *P* < 0.005) except IL-6. Similarly, correlation between was reported with *K. pneumoniae*, *P. aeruginosa*, and *E. faecalis* and microcidal proteins released. The authors suggested that 3 molecules (NAP-2, SDF-1α, and IL-6) displayed the strongest correlation with bacterial growth inhibition with all strains of bacteria tested. The concentration of microcidal protein concentrate was higher in L-PRP compared to L-PRP cryo and PRP. The authors concluded the in vitro antibacterial effectiveness of L-PRP, PRP and of cryo-preserved L-PR. The strongest correlation was observed between microbial activity against *S. aureus*, *K. pneumoniae*, *P. aureus*, and cytokines NAP-2, SDF-1α, and IL-6 although significant correlation was observed with other cytokines tested.

Lu et al. assessed chitosan–gelatin sponge (CSGT) as a vehicle to deliver PRP [36]. The authors were able to show that CSGT had good thermostability and mechanical properties as well as efficient water absorption and retention capacities. The in vitro study showed that CSGT effectively inhibited the growth of *E. coli* and *S. aureus*, and CSGT healed wounds quickly in animal studies. Additionally, acceleration of wound healing was observed in CSGT loaded with PRP. The authors suggested that CSGT and CSGT with PRP were suitable for applications as wound dressings and may have potential for use in various biomedical applications.

Bayer et al. assessed the influence of PDGF on hBD-2 antimicrobial peptide in human primary keratinocytes and the influence of Vivostat™ PRF on hBD-2 expression in experimentally generated skin wounds in vivo [21]. Primary human keratinocytes were stimulated for 24 h with different concentration of PDGF ranging from 1:10 dilutions to 1:50 dilutions and hBD-2 expression was evaluated. The results showed that PDGF significantly stimulated hBD-2 expression after 24 h (*P* < 0.01) compared to control (cell culture medium) and the expression was mediated through activator protein 1 pathway. The authors also have shown that PDGF stimulated primary keratinocytes to produce IL-6 as early as 4 h. To analyze the influence of Vivostat™ PRF on the hBD-2 expression, wounds were treated with Vivostat™ PRF. The result showed that expression of hBD-2 was significantly increased by Vivostat™ PRF compared to control (treated with NaCl 0.9%). The authors conclude that hBD-2 induction by thrombocyte concentrates could contribute positively to chronic and infected wound healing.

Bayer et al. assessed the influence of PDGF on primary keratinocytes proliferation by measuring the expression of Ki-76, a marker for cell proliferation (N = 10) [34]. PDGF caused a significant decrease in Ki-67 expression in a time-dependent manner, independent of the epithelial growth factor receptor (EGFR) or IL-6-R pathways, suggesting reduced cell proliferation. The study concluded that topical therapy using thrombocyte concentrate lysate as PDGF or Vivostat™ PRF enhances wound healing but that was not based on enhanced keratinocyte proliferation.

Cetinkaya et al. investigated the antimicrobial effect and wound healing potential of PRF in rat model (N = 72) of MRSA bacteria [44]. A superficial wound was created, and infection was induced by injecting 0.1 mL (3 × 10^8^ CFU/mL) of MRSA. PRP was embedded within the cavity within 5 min of MRSA incubation. The inflammation score was significantly reduced when PRP, vancomycin, and vancomycin + PRP groups were compared with the MRSA group (*P* < 0.001, *P* = 0.04, and *P* = 0.04, respectively); however, vancomycin + PRP was found to be most effective. The authors also proposed that vancomycin + PRP might have a synergistic effect, and concluded that PRP alone, vancomycin alone, and vancomycin + PRP increased wound healing and decreased bacterial counts.

Cetinkayaet al. demonstrated antibacterial activity of PRP against MRSA, vancomycin-resistant Enterococcus sp. (VRE), extended and spectrum, beta lactamase producing *K. pneumoniae*, and carbapenem-resistant *P. aeruginosa* (N = 10) [45]. The bacterial strains were isolated from the deep wound tissue of patients. In the PRP group there was a 9.4- and eightfold increase of platelet count and WBC count compared to whole blood. The study showed that both PRP and PPP significantly suppressed growth of MRSA, *K. pneumoniae*, and *P. aeruginosa* as early as 1 h (*P* < 0.005) and the effect was persistent up to 10 h compared to control. The effect of PRP against MRSA and *P. aeruginosa* was significantly higher compared to PPP. PRP and PPP showed limited activity against VRE.

Cieslik-Bielecka et al. evaluated the antibacterial effect of L-PRP against selected bacterial strains (MRSA, MSSA, extended spectrum beta-lactamase, *E. coli*, *K. pneumoniae*, *E. faecalis*, and *P. aeruginosa*) in vitro, and correlated antimicrobial effect with leukocyte and platelet counts (N = 20 healthy males) [46]. The result showed that L-PRP was activated using different concentrations of thrombin and calcium chloride, and the activated products were tested in following groups: G1, 20 μL of L-PRP and 5 μL of autologous thrombin (gelatinous mass); G2, 20 μL of L-PRP and 2 μL of autologous thrombin (gelatinous mass); G3, 25 μL of liquid L-PRP; G4, 25 μL of autologous thrombin; G5, 20 μL of L-PRP and 5 μl of bovine thrombin in a calcium chloride solution; G6, 20 μL of L-PRP and 2 μL of bovine thrombin in a calcium chloride solution; and G7, 25 μL of bovine thrombin in a calcium chloride solution. The results did not demonstrate a statistically significant correlation between antibacterial effect of L-PRP and platelet count. However, there was a significant correlation between leukocyte subtype and antibacterial effect of L-PRP. Overall, L-PRP exhibited leukocyte subtype mediated in vitro antibacterial activity against MRSA, MSSA, *E. faecalis*, and *P. aeruginosa*, but no antibacterial effect was demonstrated for *E. coli*, and *K. pneumonia*.

Li et al. evaluated the potential mechanism underlying the effect of PRP when used in diabetic foot ulcer in vitro model. In this study, the diabetic foot ulcer model was created by infecting HaCAT human keratinocytes with *S. aureus* (10–10^4^ CFU/mL) and co-cultured in high glucose condition [29]. The whole blood was collected from diabetic patients without active sign of infection and any coagulation disorder and processed further to produce PRP. The study used extract of PRP (EPG) without activation by activators and PRG which was produced by centrifuging PRP and by activating using calcium gluconate/thrombin to produce PRG. The result showed that with co-culture of HaCAT and *S. aureus*, there was significant decrease in cell proliferation of HaCAT cells, suggesting that *S. aureus* impeded normal cell proliferation. The study found that both EPG and PRG significantly reduced bacterial count compared to PPP; however, after 36 h there was no difference among PRP, EPG, and PPP. EPG (20%) protected HaCAT cells from damage caused by *S. aureus* and promoted cell proliferation and the observed effect was very much dependent upon the concentration of EPG (60% did not show similar response). It was shown that EPG significantly reduced PDCD4 and NF-ĸB expression and prevented nuclear translocation of p65 protein as compared to control. IL-6 and TNF-α were significantly increased in HaCAT cells transfected with bacteria and EPG reduced the expression of IL-6 and TNF-α, suggesting overall inhibition of inflammatory response.

Knafl et al. evaluated the release of amikacin, teicoplanin, or polyhexanide from a PRF layer (N = 10) [33]. PRF was prepared using whole blood collected from 5 donors using Vivastat™ PRF and delivered in a patch containing amikacin, teicoplanin, and polyhexanides. PRF with amikacin or teicoplanin inhibited growth of *S. aureus*, *P. aeruginosa*, and *K. pneumoniae* until day 6 and increased wound healing due to slow release of amikacin and teicoplanin compared to control. PRF plus polyhexanide did not inhibit the bacterial growth, and the authors suggested that use in combination of PRF with polyhexanide is not recommended.

Różalski et al. assessed the killing effect of platelets against planktonic and biofilm cultures of *S. aureus* and tested the synergistic effect of PRP with different antibiotics (oxacillin, vancomycin, and linezolid) [31]. The authors used expired platelet concentrates (N = 5, within 1–3 days after the expiry date) and divided them in the following experimental groups: a) a suspension of unstimulated cells, b) a suspension of platelets after adenosine diphosphate (ADP) stimulation, c) and d) cell lysates prepared from unstimulated and stimulated cells, respectively. The authors used ADP to activate platelets instead of thrombin. The authors reported that “expired” platelets and their lysates significantly reduced the population of *S. aureus* and also decreased metabolic activity of biofilm formation, suggesting that even after the expired time for transfusion (total time 6–8 days of life), platelets maintain significant microbicidal activity. The authors also claimed that antibacterial activities were enhanced after activation with ADP compared to unstimulated platelets. Platelet lysates showed a synergistic effect with oxacillin and vancomycin but not with linezolid, suggesting interference with the cell wall synthesis.

## Preclinical literature

Nimal et al. assessed the efficacy of tigecycline nanoparticles loaded into chitosan-PRP hydrogel in inhibition of *S. aureus* growth [47]. Tigecycline and tigecycline nanoparticle-incorporated chitosan gel exhibited antibacterial activity against *S. aureus*. The authors concluded that the gel system could serve as an effective medium for antibiotic delivery when applied on the infection sites to effectively forestall various skin infections caused by *S. aureus*.

Shibata et al. evaluated the effectiveness of controlled release of PRP from biodegradable gelatin hydrogen using a rabbit ischemic sternal model. PRP was prepared using the whole blood from Japanese white rabbits (N = 16) [37]. The rabbits were randomized to 4 groups: (a) 300 µL of PBS, (b) 300 µL of PRP solution, (c) 30 mg of gelatin hydrogel incorporating 300 µL of PRP (PRP + hydrogel), and (d) control group (no treatment). The results showed that the fibrotic area ratio with fracture area was significantly higher in the PRP + hydrogel group (22.7%, 95% confidence interval [CI] 12.03–33.27) compared to control (11.4%, 95% CI 12.03–33.27) and PRP alone (13.2%, 95% CI 8.81–17.51). Bone regeneration was further investigated using osteocalcin staining. The osteocalcin staining was significantly higher for the PRP + hydrogel group (17.3%, 95% CI 12.74–21.83) than the control or PRP alone group (*P* < 0.05). The authors concluded the controlled release of PRP using hydrogel might be an effective way to enhance sternal healing.

Yassin et al. compared the efficacy of PRP wafers and PRP powder in terms antibacterial and healing effects using in vitro and ex vivo animal models [48]. The authors used blood collected from consented patients to prepare PRP, and PRP was further processed to prepare lyophilized PRP powder using freeze drying (Christ, Alpha 2–4 LD plus) and PRP wafers using sodium carboxymethylcellulose. The authors reported that wafers maintained the desired appearance of a wound dressing and displayed the stable storage characteristics as revealed by scanned electron microscopy. The platelet count was similar in all PRP products: PRP (1.5 × 10^6^ platelets/µL), lyophilized PRP powder (1.6 × 10^6^ platelets/µL), and PRP wafers (1.7 × 10^6^ platelets/µL), suggesting that lyophilization did not affect platelet count. All PRP products exhibited antibacterial effect against *A. baumannii*, suggesting the different formulations did not compromise the activity of PRP. Both lyophilized power (*P* < 0.0002) and PRP wafer (*P* < 0.0001) had a better healing effect as suggested by the wound size and re-epithelization of the wound healing from day 1. The authors concluded that PRP wafers showed the desired characteristics in terms of the water loss percentage, platelet count, content uniformity, and hydration and provided better results than lyophilized PRP powder in an antimicrobial efficacy test, wound size measurements, and histopathological analysis. The authors suggested that PRP wafers might offer an effective pharmaceutical delivery system for the application of PRP to a wound area.

Ikono et al. used chitosan-PRP nanoparticles to improve the viability of PRP and prolong release of growth factors [49]. The results demonstrated that chitosan-PRP nanoparticles had strong antibacterial activity against *Streptococcus mutans* (*S. mutans*) (90.63% inhibition), suggesting a novel mechanism to deliver PRP in wounds to promote healing.

Farghali et al. compared treatment with autologous PRP prepared with the double spin method, to treatment with topical clindamycin in MRSA infected, full thickness cutaneous wounds [50]. Wounds 30 mm in diameter (9 mm^2^ in area) were created on the thoracic region in 6 dogs. The wounds were inoculated with MRSA isolated from a naturally infected wound in a non-experimental dog. The control group (n = 3) was treated twice daily with topical clindamycin and the experimental group (n = 3) received a subcutaneous injection of 3 mL calcium chloride-activated autologous PRP prepared by the “2-spin” method once each week on days 7, 21, and 28. Calcium chloride-activated PRP inhibited the growth of MRSA in vitro at a dilution of 1:4 in the sample taken before conducting the experimental infection. The minimum inhibitory concentration (MIC) against MRSA revealed a pattern of fourfold increases; it reached 1:16 after 1 week of treatment with PRP and continued increasing through the second week of treatment to inhibit the growth of MRSA at 1:64 in the third week of treatment. In contrast, the non-calcium chloride–activated PRP in which platelets did not release biologically active components did not show any inhibitory effect.

After 1 week of infection, the wound area had reached 93.0 ± 4.4 mm^2^ in the control group and 93.0 ± 1.7 mm^2^ in the PRP-treated group. After 1 week of PRP treatment, the wound size was smaller in the experimental group than in the control group. The wound size at week 1 was 24.1 ± 1.6 mm^2^ in the control group and 8.6 ± 0.7 mm^2^ in the PRP-treated group. At the 2nd week, the wound size in the control group was 25.0 ± 10.6 mm^2^ while that in the PRP treated group was 2.2 ± 0.2 mm^2^. At the 3rd week, the wound size was 5.3 ± 2.9 mm^2^ in the control group and 0.5 ± 0.2 mm^2^ in the PRP-treated group. A significant size reduction (*P* < 0.05) was found after 1 week of treatment. The wound contraction percentage was elevated (*P* < 0.05) in the PRP-treated group compared to the control group at all intervals, with a significant elevation at week 1. The re-epithelization rate percentage was significantly increased in the PRP-treated group at week 2.

The PRP experimental group demonstrated superior healing by all measures: bacterial counts from wound biopsies decreased significantly over time. Expression of TNF-α and VEGF-A genes were increased in the wound tissue of the PRP group versus the control group, as was the concentration of malondialdehyde and glutathione reductase. Clinical examination/measurement, clinical examination, bacterial growth evaluation, biochemical assessment of oxidative stress, quantification of the expression of growth factor and cytokine genes, histopathological analysis, and immunohistochemical evaluation all suggested that PRP had a strong effect on MRSA; however, notably this effect was only observed when the PRP was activated with calcium chloride.

## Clinical studies

Dorge et al. investigated the use of PRP in high-risk patients undergoing cardiac surgery with full sternotomy [5]. Patients qualified as high risk by having at least one of the following risk factors: diabetes mellitus (oral antidiabetic or insulin-dependent), chronic obstructive lung disease (inhaled steroids), renal insufficiency (chronic dialysis), obesity (body mass index > 30 kg/m^2^), left ventricular function (ejection fraction < 35), age > 80 years, use of double internal mammary artery, or chronic use of systemic corticosteroids. After giving informed consent, patients were prospectively randomized to sternal application of PRP (n = 97) or to the control group (n = 99) that received standard wound care. Both groups received prophylactic 3 × 2 g cefazolin intravenously (IV) after induction of anesthesia. PRP was prepared and simultaneously injected in sternal edges along with thrombin using recommended dual spray applicator. The results showed that the use of PRP (n = 6, 6.2%) did not reduce the incidence of DSWI compared to the control group (n = 3, 3.0%) (*P* < 0.293). The authors concluded that local application of PRP in cardiac surgery patients with full sternotomy at high risk for sternal complications did not reduce the incidence of DSWI.

Serraino et al. retrospectively evaluated whether PRP application inside the sternotomy wound after sternal closure can prevent sternal wound infections (both superficial sternal wound infection and DSWI) (N = 1093) [32]. In the study, PRP following activation with thrombin and 10% calcium chloride was applied to the sternal region before closure of the subcutaneous tissues. Patients in the control group underwent sternotomy without PRP and received standard care. Patients in both control (n = 671) and PRP (n = 422) groups received prophylactic teicoplanin (400 mg/day IV) and ciprofloxacin (2 × 400 mg/day IV) until postoperative day 5, and were followed up at 1 week and 1, 6, and 12 months postoperatively. The result showed that 0.2% of patients developed DSWI in the PRP group versus 1.5% in the control group (*P* < 0.043), while the incidence of surgical site infection (SSI) was 0.5% in the PRP group versus 2.8% in control group (*P* < 0.006). The authors concluded that PRP effectively reduces the incidence of both SSI and DSWI in sternotomy patients.

Patel et al. assessed the addition of PRP to standard wound care in all patients undergoing sternotomy for cardiac surgical procedures (N = 2000) [4]. The data were collected prospectively from the patients undergoing open cardiac surgery requiring sternotomy and analyzed retrospectively. PRP was prepared using FDA-approved Magellan® Autologous Platelet Separator System (Arteriocyte Medical Systems) and was activated using thrombin and calcium chloride. There was no significant difference in patient demographics in PRP group (n = 1000) versus control group (n = 1000), except there were more ventricular assist device implants/heart transplants patients in the PRP group. The authors reported that the use of PRP reduced the incidence of DSWI from 2.0 to 0.6%, surgical wound infection from 8.0 to 2.0%, and the readmission rate from 4.0 to 0.8%. The authors also demonstrated cost/benefit of using PRP in DSWI and surgical infection prevention. The use of PRP reduced the costs associated with the development of deep and superficial wound complications from $1,256,960 to $593,791, which is nearly a 50% decrease in the cost of care. The authors concluded that PRP decreased the incidence of sternal wound complications following cardiac surgery.

Wozniak et al. qualitatively assessed microbial flora in venous leg ulcers following single intradermal injection with PRP injected to ulcer margin [51]. The study was uncontrolled (no positive or negative control) for the bacterial plating. The study showed that PRP therapy significantly improved healing in 61.8% of subjects (N = 34). The microbial analysis identified 81 varieties of microbes and the majority of cultures from a single swab from the patient (73.5%) showed the presence of multiple species. Gram-positive bacteria were isolated from over 30% of patients and gram-negative bacteria from 59%, with anaerobic bacteria and fungi making up 9.6% and 1.2%, respectively. The most commonly isolated gram-positive bacterial species included *S. aureus*, *E. faecalis*, *and Bacteroides fragilis* (*B. fragilis*), and the most commonly reported gram-negative bacterial strains were non-fermenting bacilli, *P. aeruginosa*, *A. baumannii*, and *Stenotrophomonas maltophilia*, Enterobacteriaceae, *Serratia marcescens*, *Morganella* (*M*) *morganii*, *E. coli*, *P. mirabilis*, *K. oxytoca*, and *E. cloacae*. The study reported that there were increased numbers of isolates after PRP treatment for MSSA, Streptococcus group B, *M. morganii*, *E. coli*, *P. mirabilis*, *K. oxytoca*, *E. cloacae*, *P. aeruginosa*, *Bacteroides fragilis*, *Prevotella denticola*, and *Candida albicans*; however, the authors observed a visible reduction in overall plated bacterial colonies in about half the patients. PRP therapy showed a marked increase in the isolation ratio of MRSA and *E. coli*. The study concluded that local application of PRP on the surface of venous ulcer reduces the number of colonies and, in contrast, also contributed toward an increased variety of bacterial flora in some cases.

In a retrospective study, Hamman et al. evaluated the topical application of autologous platelets concentrate and vancomycin in preventing DSWIs in patients undergoing a cardiac surgical procedure with full sternotomy, and who had not previously undergone coronary artery bypass, graft or value surgery or other procedures requiring sternotomy (N = 1866) [3]. The patients received prophylactic antibiotics in accordance with national guidelines 1 h before and 48 h after the procedure. Following activation with 5 mL of 10% calcium chloride, PRP was mixed with 2 g of vancomycin hydrochloride powder and the paste was applied to the edges of the sternum just before closure. The investigators reported that incidence of DSWI was significantly decreased by PRG (n = 548) compared to historical controls who did not receive the PRG (n = 1318). During the study 4 patients in the control group developed severe DSWI 4 months after the surgery while no patients in the experimental group developed DSWI.

Englert et al. examined the effect of PRP (n = 30) vs. platelet poor plasma (PPP, n = 15) on postoperative sternal wound infection and evaluated pain reduction (chest and leg pain), amount of decreased bruising area, and platelet indices under preoperative and postoperative conditions [52, 53]. PRP was prepared by the Magellan Autologous Platelet Separator System (Medtronic) that increased platelet concentration by almost 5 times and was activated using calcium chloride and thrombin. The results suggested that the application of PRP before the closure of the leg incision after the saphenous tissue harvest, and before sternum wiring results in decreased chest pain and leg pain as early as day 1 as compared to PPP control group.

Tran et al. evaluated the effects of activated PRP on diabetic foot ulcer healing (N = 6) [54]. All patients had non-healing foot ulcers and multiple comorbid conditions. The authors used calcium chloride to activate both PRP and PPP. Activated PRP was applied as fibrin gel in the wound, activated PPP was injected in the diabetic foot ulcer from days 4 to 8, and patients were monitored for 12 weeks. The results showed that 100% (6/6) of the ulcers completely closed after about 7 weeks, and no adverse events were reported. The authors concluded that activated PRP injection was an effective method to treat the non-healing foot ulcers.

Vang et al. randomized 38 patients to receive either autologous platelet gel or standard care to treat the sternum wound and the saphenous vein harvest site after coronary artery revascularization [55]. The authors evaluated postoperative pain, discoloration/bruising, and surgical site infection. All patients had multiple comorbid conditions and comorbidities were similar between groups. In the treatment group, 87% of patients experienced less pain on the sternum on postoperative day 1 versus 67% in the control group. No patient experienced either superficial or deep sternal wound infection; however, one patient in each group was diagnosed with infection at the saphenous vein harvest site. Patients differed in postoperative wound care and platelet count and the study was underpowered so the authors could not assign outcomes to a particular therapy.

## Discussion

Cutaneous wound healing comprises 3 distinct phases: inflammation, proliferation, and maturation. In an infected wound, the combination of bacterial endotoxins, proteolytic enzymes, release of growth factors and metalloproteinases causes aggravated inflammation, thereby affecting the cellular machinery needed for cell proliferation and wound healing [56]. The resolution of the inflammatory response is essential for completion of the cycle and for successful wound healing.

Bacterial infection of cutaneous chronic wounds is a serious, life- and limb-threatening complication, impairing wound healing and tissue regeneration and potentially leading to septic shock. Host status, blood glucose level/diabetes, albumin levels/nutritional state, age, and body mass index are just a few of the risk factors that contribute to increased risk of chronic wound infection. Standard care of infected wounds includes debridement, relief of pressure, application of antiseptics, various anti-infective dressings, hyperbaric oxygen therapy, negative pressure wound therapy, and antibiotics. Standard care therapies are effective in most wounds; however, a minority are recalcitrant to these therapies. In addition, the evolution of antibiotic-resistant organisms such as MRSA has prompted clinicians to seek an alternative/adjunctive method for treatment and prevention of wound infection [2].

In this systematic literature review, we aimed to (1) identify evidence supporting or refuting the efficacy of PRP as an antibacterial agent for prevention of DSWI and wound healing, and if effective, identify proposed mechanism/s, and (2) identify gaps in the evidence for an antibacterial effect of PRP. In addition to these pre-specified objectives, a post-hoc assessment of data from this SLR was conducted to identify ideal characteristics of PRP specific for cardiothoracic surgery which may impact clinical findings and guide clinical use.

The following research questions drove our analysis: Does PRP exert an antibacterial effect? What types of bacteria are affected by PRP? Is PRP bactericidal or bacteriostatic? Is there a synergistic effect of between antibiotics and PRP on bacterial killing? What is the mechanism involved in the antibacterial effect of different components of PRP, and can it be enhanced?

## Does PRP exert an antibacterial effect?

The literature gleaned in this review reveals some contradictory outcomes. Seven clinical therapeutic trials were evaluated in this review [3–5, 32, 52, 54, 55], and one trial evaluating flora isolated from chronic leg ulcers [51]. Four trials evaluated DSWI after cardiac procedures in wounds treated with either PRP or standard care. Of the four studies, one found a negative result, with no benefit observed for PRP against standard care for DSWI. The other three all had positive results; however, all studies were underpowered to rule out chance instead of a true effect of PRP. One group used an incorrect comparative statistical test for the primary outcome [32]. A retrospective cohort study by Patel et al. with a thousand patients in each group demonstrated a reduction in the incidence of DSWI from 2% in controls to 0.6% in the PRP group [4]. Post hoc power and sample size analysis (Table 8, last column) suggests that this study is adequately powered but the retrospective nature of the study and the lack of a priori sample size determination forces a consideration of selection bias. A randomized controlled trial (RCT), or larger observational study to collect real-world evidence to further evaluate PRP for the prevention of DSWI is required to confirm these findings.

**Table 8:** Power and sample size calculations (2-tailed)

	Required sample size a priori		Achieved power post hoc
Input			
Proportion p2	0.01	0.016	0.026
Proportion p1	0.03	0.006	0.1
α err prob	0.05	0.05	0.05
Power (1 − β err prob)	0.8	0.8	–
Allocation ratio N2/N1	1	1	–
Sample size group 1	–	–	1000
Sample size group 2	–	–	1000
Output			
Critical z	1.9599640	1.9599640	− 1.9599640
Power (1 − β err prob)	–	–	0.9999995
Sample size group 1	769	1707	–
Sample size group 2	769	1707	–
Total sample size	1538	3414	–
Actual power	0.8005067	0.8000975	–

z tests—proportions: difference between 2 independent proportions [4, 56]

The incidence of DSWI is relatively rare, occurring in between 1.6 and 3% of patients after cardiac procedures [57]. As a rare and serious condition, the sample size required to ensure that there is a true difference between the groups is very large. For example, to find a reduction from 3 to 1% with 80% power would require 769 patients in each group (Table 8, first column); however, 3% is a high estimate of incidence. In a cohort of 176,537 patients, Sears et al. observed an incidence of DSWI of 1.6% [57]. To find a reduction from 1.6 to 0.6% would require 1707 patients in each group (Table 8, second column).

None of the clinical studies in this review were of adequate quality to draw a conclusion for an intervention, ranging from Oxford evidence level 2b to level 4 [26]. There were multiple sources of bias, such as none of the clinical studies calculated power and sample size a priori and all except Patel et al. were underpowered to find a true difference between the groups. In addition, none were blinded, and most were retrospective; as a result, selection bias cannot be ruled out. Further, the variability in production of the PRP and protocols for administration needs to be reduced in order to draw conclusions about efficacy of this therapy.

Despite the rarity of DSWI, the large number of cardiac procedures (600,000/year in the US) and the seriousness of the diagnosis for the patient and burden to the healthcare system warrants continued intensive research on treatment options to prevent and treat it [57].

Two additional clinical studies evaluated the efficacy of PRP for saphenous vein harvest site and for diabetic foot ulcers. Both had low sample size and were poorly controlled, and thus were level 4 studies [29, 54].

The in vitro data were also somewhat contradictory in terms of efficacy against specific bacteria. Most authors agreed that platelet preparations are active in varying degrees against bacterial strains common in wounds including MRSA, MSSA, *E. coli* (extended spectrum beta lactamase), *K. pneumonia*, *E. faecalis*, *P. aeruginosa*, *B. megaterium*, *P. mirabilis*, *E. cloacae*, *B. cereus*, *B. subtilis*, *S. epidermidis*, and *A. baumannii* [15, 28, 31, 39, 40, 42]. However, when considering specific bacterial species (*P. mirabalis* and *P. aeruginosa* for example), results were inconsistent. This is in line with efficacy of specific existing antibiotics against targeted bacterial species; similarly, autologous PRP is unlikely to be a universal therapy against all bacterial species. Some authors reported that bacterial growth was inhibited only during the early period of incubation (as early as 0.5 h after treatment with PRP) with later regrowth of bacteria observed, suggesting a transient effect of PRP or the need for an additional dose as antimicrobial factors are exhausted [28, 39]. Other groups suggested that PRP did not inhibit the growth of *P. aeruginosa*, instead suggesting that it may cause an exacerbation of infection with this organism [15]. In contrast, other authors concluded that PRP inhibited the growth of this bacterium [40]. Similar observations were reported in the growth inhibition of *P. mirabalis* [23].

The contradictory findings are in part due to heterogenous methods of PRP preparation, activation, administration, and inadequate sample size and power, and in part due to the heterogeneity of the target for treatment. In addition, the PRP produced for the bench experiments was isolated from healthy subjects, which may not correlate with PRP isolated from individuals with comorbidity and infection. There are multiple strains of bacteria in the wound environment, frequently resulting in polymicrobial infections, and successful treatment depends upon the type of wound and host status. Calcium chloride is the most commonly utilized activator but several studies reported other methods of activation such as freeze/thaw, bovine thrombin, autologous thrombin, or calcium gluconate. Differences in PRP processing methods and the lack of a standardized protocol for optimal yield of platelets, leukocytes, and various cellular components and antimicrobial proteins introduce confounders and heterogeneity that make it difficult to accurately assess efficacy.

## What types of bacteria are affected by PRP?

Despite the heterogeneity in the studies in terms of PRP preparation, treatment targets, and experimental methods, most studies consistently show that PRP is most effective against gram-positive bacteria, including the difficult to treat gram-positive species MRSA [15, 39, 40, 43, 50]; however, some authors have shown activity against gram-negative species such as *E. coli* [25, 30]. Perhaps the most well-designed and compelling study in this review to demonstrate efficacy of PRP against MRSA was the canine study by Farghali et al. [50]. This study found a remarkable improvement in MRSA-infected wound healing with a number of well-controlled measures versus the control group treated with clindamycin.

## Is PRP bactericidal or bacteriostatic?

PRP is both bactericidal and bacteriostatic. Depending on the bacterial load, host status, bacterial type, and the overall “dose” of PRP, it may achieve the MIC and overcome the rate of bacterial growth enough to stop replication. If the dose of PRP is insufficient, it may slow growth but be subsequently overcome as the antimicrobial aspects of PRP are depleted over time. Several authors suggested a continued dose of PRP over the wound healing time is more effective than a single application [50].

## Is there a synergistic effect between antibiotics and PRP on bacterial killing?

There is weak preclinical evidence suggesting that when used as an adjunct to antibiotics, PRP may have a synergistic effect; however, other studies contradict this [3, 46]. Platelet lysates showed a synergistic effect with β-lactam antibiotic (oxacillin) and glycopeptide (vancomycin) but not with oxazolidinone (linezolid) [31]. Bielecki et al. describe a subset of platelet antimicrobial proteins defined as classical chemokines with direct antimicrobial properties that also act in consort with conventional antibiotics and are less prone to inducing bacterial resistance [38]. In another study, Bielecki et al. showed that L-PRP gel antimicrobial properties could be enhanced by antibiotics. It is unclear if there is true synergism or simply multiple avenues of bacterial attack. Platelets are also angiogenic, and the formation of new blood vessels at the wound site may facilitate antibiotic delivery and deliver native blood supply that can assist in healing.

## What is the mechanism involved in the antibacterial effect of different components of PRP, and can it be enhanced?

The role of native platelets in wound healing, inflammation, and antibacterial effect is well established [7, 10, 58]. Li et al. and other authors describe the multiple roles of native platelets in host defense against infection including to: 1) generate antimicrobial oxygen metabolites; 2) facilitate complement fixation on bacteria; 3) internalize and clear pathogens from the blood stream; 4) execute antibody-dependent cell cytotoxicity; 5) potentiate antimicrobial mechanisms of leukocytes; and 6) degranulate and release a variety of cationic antimicrobial peptides such as VEGF, PDGF-BB, IGF-1, and TGF-β1 [7, 10, 29, 41, 42, 58].

It is a reasonable hypothesis that supraphysiologic platelet concentration at the wound site might facilitate healing and prevent infection; however, whether PRP contains all of the constituents present for native platelets in vivo that are necessary for activation, and both direct and indirect bactericidal function, is not well understood. PRP preparation is a complex mixture of platelets, WBC, plasma, and soluble factors (cytokines and growth factors) that various authors have hypothesized may be responsible for the antimicrobial activity of platelet preparations; however, the exact role of each component, or of multiple components in combination, remains poorly understood. Numerous groups have proposed multiple mechanisms that may contribute to the antibacterial effect of platelet preparation, including release of platelet antimicrobial proteins, plasma complement and complement-binding proteins, peptides of the innate immune defense, increased concentration of different growth factors, and increased ROS in response to bacterial LPS. However, there is no consensus on the active constituents and how these components interact to contribute to antibacterial and wound healing properties.

Leukocytes within PRP are also involved in direct bacterial killing, and in antigen-specific immune response but they are not strictly necessary for PRP bactericidal effect. Platelets augment the antimicrobial functions of leukocytes but have an independent bactericidal function as well [58, 59]. One group found no significant difference in antibacterial activity between PRP-LP and PRP-HP preparations despite substantial differences in platelet and WBC counts [28]. Similar observations were reported by other authors who found no correlation between antimicrobial activity and the concentration of platelets and leukocytes. In contrast, Cieslik-Bielecka et al. suggested that a strong relationship was observed among selected leukocyte subtypes (T and B lymphocytic NK cells, monocytes, and granulocytes with CD45) with antimicrobial activity of L-PRP [46, 60]. Other authors suggested that PRP significantly increased the proliferation and migration of fibroblasts, indicating a role of PRP in regeneration of damaged tissue [47]. Some authors have suggested that inclusion of WBCs in PRP may help to improve the stability of the scaffold and increase the antimicrobial potential [60]. However, the results from the study by Bielecki et al. showed that a higher leukocyte dose did not significantly improve the antimicrobial properties of PRP. It has also been suggested that additional leukocyte content might increase the inflammatory response at the site because of the metalloproteases, pro-inflammatory proteases, and acid hydrolases secreted by WBCs [38]. In addition, platelet preparations are shown to increase the concentration of different growth factors such as PDGF, TGF-β1, VEGF, IGF-1, IL-6, IL-8, EGF, and IL-1β that promote the wound healing process [24, 29, 31].

Based on the results from these studies and other literature, it appears that leukocyte-rich PRP preparations theoretically have enhanced antibacterial activity induced by multiple factors, including the presence of a rich source of antimicrobial molecules (eg, defensins, lysozyme, myeloperoxidase) but a definitive mechanism, and how this might be translated into clinical medicine in terms of preparation and dosing, is not currently understood [23–25, 31, 39, 40].

Another proposed mechanism relates to the increase in the concentration of MPO by PRP, indicating WBC activation, but there is no correlation of MPO release and cell count [23, 24]. It has also been proposed that MPO is released at higher bacterial load and is not a first-line defense mechanism at lower bacterial concentrations, which might be more relevant to the use of PRP for infection prophylaxis. Tohidnezhad et al. suggested that the increased release of hBD-2 and hBD-3 after PRP activation may act as a first-line defense by binding with negatively charged bacterial cell walls and generating pores leading to bacteria inhibition [40]. A study by Aktan et al. showed that LPS and LTA have no effect on platelet superoxide production or heterotypic aggregate formation [25]. It has been suggested that direct interaction of platelets with bacteria releases ROS that cause ROS-dependent cell cytotoxicity of bacteria as a potential mechanism of antibacterial effect of PRP [7]. Even after the end of shelf-life (typically 5–7 days after collection), platelets are suggested to be a good source of antimicrobial low molecular weight proteins that exhibit antibacterial effect [31]. A study by Mariani et al. reported increased concentrations of soluble factors (MIP-1α/CCL3, RANTES/CCL5, GRO-α/CXCL1, NAP-2/CXCL7, IL-8/CXCL8, SDF-1α/CXCL12 and IL-6) that were considered strongly correlated to bacterial growth inhibition [14]. PRPG significantly increased the proliferation and migration of fibroblasts, suggesting the role of PRP in wound healing.

PRP has gained attention in the last two decades due to widespread off-label clinical use based on the hypothesized regenerative potential and antibacterial effect, although the exact mechanism, dose, and efficacy of PRP activity against different strains of bacteria is not established. As such, the foundational work that would provide evidence to support a specific treatment indication is incomplete. To date, there is no standardized preparation method, standardized dose, or validated method or protocol for administration of PRP that would allow translation into well-designed and adequately powered clinical trials to demonstrate efficacy.

There are several of PRP separation systems are on the market. These systems are approved to collect and manufacture PRP, although none are approved for a specific therapeutic indication.

## How does the existing data guide clinical use of autologous PRP in cardiothoracic surgery?

While there isn’t solid evidence to support what may be the ideal autologous PRP product and treatment protocol for cardiothoracic surgery to prevent surgical site and deep sternal wound infections due to clear foundational data gaps, there are clear data trends to guide clinical use. Autologous PRP can be prepared rapidly in a consistent fashion, in the operating room at patient’s bedside and has been shown to be safe for clinical use. In the study conducted by Patel et al., the authors highlighted that the reduced rate of infection can be attributed to the faster healing of wounds with PRP application. The authors also mentioned the limitation of sample size and thus a powered conclusion of this mechanism [4].

Currently there are two FDA-approved platelet-derived products for the treatment of healing wounds but neither is indicated as an antibacterial agent. One product, Procuren, is no longer manufactured and was an autologous platelet-derived growth factor marketed for treatment of chronic, non-healing wounds. The other product, Becaplermin (Regranex wound gel), is a recombinant platelet-derived growth factor approved via a Biologics License Application for treatment of neuropathic ulcers in conjunction with standard wound care. There is excellent foundational basic science and four well-designed and powered RCTs demonstrating efficacy in wound healing for Regranex. As a result, reimbursement for this therapy is covered by CMS and private payers. Procuren was considered investigational and was never covered by insurers. 

## Conclusion and areas for future research

Autologous PRP therapy may be effective in treating and preventing wound infection but the basic science as well as the clinical literature is conflicting. There is no definitive answer to the questions posed in this review, resulting in identification of multiple areas for further research. Providing a supraphysiological “dose” of platelets may add to the natural function of native platelet response. It is important to define and quantify the other constituents necessary in the wound healing milieu and ensure that they are present in adequate amounts to simulate platelet autocrine and paracrine factors.

A number of gaps in foundational knowledge must be bridged prior to proposing a well-designed and statistically powered clinical trial:

1. Similar to Farghali et al. [50]: Quantitate constituents of PRP from the Terumo system that are known/well-established to enhance wound healing; i.e., endogenous platelet-derived growth factor promotes the chemotactic recruitment and proliferation of cells involved in wound repair, enhancing the formation of granulation tissue.
2. Standardize and validate PRP input and output from the Terumo system to achieve adequate concentrations of one or more active wound healing factors for efficacy and determine correlation of these data with complete blood count results in the individual. Some populations with comorbidities may be inappropriate for autologous PRP therapy. All of the in vivo studies used healthy donors, and many pooled PRP from multiple donors. There were no inclusion/exclusion criteria in most cases, and no reporting of donor demographics or controlling for any other potential confounders.
3. Standardize and optimize activation of PRP: This review indicates that activation with calcium chloride is necessary to achieve any antibacterial activity of PRP.
4. Dosing: Optimize MRSA MIC with the standardized PRP output and determine the duration of activity to understand how many applications are required for either prevention or treatment.
5. Translate the in vitro data to preclinical testing for efficacy similar to Farghali et al. [50] and include bioavailability testing.
6. Translate the preclinical in vivo animal data to a pilot clinical trial, and then design and conduct one or more well-designed and adequately powered RCTs.

This systematic literature review and appraisal revealed specific considerations to guide clinical use/misuse of autologous PRP in cardiothoracic surgery and prevention of deep sternal wound infection and surgical site infections, despite the above-mentioned clear gaps in foundational knowledge. Namely:

1. PRP preparation and use.
  - Use an FDA-approved Autologous Platelet Separator System for cell collection, separation, and preparation.
  - Standardize and optimize activation of PRP with appropriate activation agents (Calcium chloride/Thrombin combination).
  - Optional: Mix activated PRP with vancomycin hydrochloride powder.
    Note: Although the addition of vancomycin has shown positive results [3]. To the best of our knowledge, the additive benefit of antibiotics over PRP has not been studied in a powered study.
  - Following activation of the PRP, apply the paste directly to the edges of the sternum immediately before closure.
2. Clinical protocol for use.
  - Patients who undergoing cardiothoracic surgery with sternotomy who are at high risk of developing a surgical site or deep sternal wound infection (eg, diabetes mellitus, previously undergone coronary artery bypass, graft or value surgery or other procedures requiring sternotomy, renal insufficiency, obesity, left ventricular function, age > 80 years, use of double internal mammary artery, immune compromised or suppressed) may benefit from autologous PRP therapy which has a favorable risk/benefit ratio in this population.
  - Topical post-surgical PRP application should be combined with IV infusion of antibiotics prophylactically and up to 5 days postoperative (teicoplanin, ciproflaxcin, and/or vancomycin, in accordance with national guidelines and per clinical judgement).

Overall, the quality of the clinical trials in this review is low, and collectively qualify as Oxford level C. There is marked bias, a lack of statistical power and repeatability, and the findings are ambiguous. The preclinical and bench data are more compelling, particularly the study by Farghali et al. [50]. There may be sufficient published data to define future steps (see above) necessary to validate PRP as a therapy with a specific indication. The recommendation is to focus future research on PRP activity against MRSA for the following reasons: the evidence for a bactericidal effect of PRP on MRSA is stronger than for other bacterial species, there is a great clinical need given the threat of developed resistance to vancomycin, MRSA is common in chronic wounds and is difficult to treat effectively, requiring extensive periods of time on IV antibiotics. Most infected wounds are polymicrobial and the addition of PRP as an adjuvant to standard wound care and to broad spectrum antibiotic therapy may be advantageous in the treatment of MRSA and the prevention of vancomycin resistance. Despite the lack of well-designed prospective RCTs with a narrow confidence interval, the current bench and clinical data suggest that there may be benefit to the use of PRP as an adjunct to standard care for prevention of DSWI (and this may extend to other wound types). Given that there is very little risk in autologous PRP, the risk/benefit ratio is favorable. Treatment or prevention of infection with PRP is promising but there is a need for foundational bench and preclinical animal research to optimize PRP as an antibacterial agent, and to provide data to aid in the design and conduct of well-designed RCTs with adequate power to confirm antimicrobial efficacy of PRP in specific disease states and wound types. Specifically, future research should focus on filling foundational gaps identified above in order to completely understand the promising impact of this therapy on clinical outcomes.

## Data Availability

Not applicable.

## Abbreviations

*A*: *Acinetobacter*
ADP: Adenosine diphosphate
AGF: Autologous Growth Factor filter™
*B*: *Bacillus*
BC-PRP: Buffy-coat platelet-rich plasma
*B. fragilis*: *Bacteroides fragilis*
CFU: Colony formation units
CI: Confidence interval
CSGT: Chitosan–gelatin sponge
DSR: Distiller Systematic Review
DSWI: Deep sternal wound infection
*E. cloacae*: *Enterobacter cloacae*
*E. coli*: *Escherichia coli*
ECS: Electa Cell-Separator™
*E. **faecalis*: *Enterococcus faecalis*
EGF: Epithelial growth factor
EGFR: Epithelial growth factor receptor
EPG: Extract of PRP
GPS: Gravitational Platelet Sequestration System™
hBD-2, hBD-3: Human beta defensin-2, -3
IGF: Insulin-like growth factor
IV: Intravenously
*K*: *Klebsiella*
L-PRP: *Leukocyte and platelet-rich plasma*
LPS: *E. coli*-derived lipopolysaccharide
LTA: *S. aureus*-derived lipoteichoic acid
*M*: *Morganella*
MIC: Minimum inhibitory concentration
MPO: Myeloperoxidase
MRSA: Methicillin-resistive *Staphylococcus aureus*
MSSA: Methicillin-sensitive *Staphylococcus aureus*
*P. acnes*: *Propionibacterium acnes*
*P. aeruginosa*: *Pseudomonas aeruginosa*
PBS: Phosphate-buffered saline
PDGF: Platelet-derived growth factor
PDGF-BB: Platelet-derived growth factor-BB
PEF: Platelet electric field
PG: Platelet gel
PLG: Platelet leukocyte gel
PLG-AT: PLG with autologous thrombin
PLG-BT: PLG with bovine thrombin
*P. mirabilis*: *Proteus mirabilis*
PPP: Platelet-poor plasma
PRF: Platelet-rich fibrin
PRG: Platelet-rich plasma gel
PRGF: Platelet-released growth factor
PRP: Platelet-rich plasma
P-PRP: Pure PRP
PRP-HP: PRP with high platelets
PRP-LP: PRP with low platelets
RBC: Red blood cell
RCT: Randomized controlled trial
ROS: Reactive oxygen species
*S*: *Staphylococcus*
S/D-PL: Solvent/detergent-treated and virally inactivated platelet concentrate
*S. mutans*: *Streptococcus mutans*
SSI: Surgical site infection
TGF-β1, TGF-β2: Transforming growth factors beta
TRAP: Thrombin receptor-activating peptide
VEGF: Vascular epithelial growth factor
VRE: Vancomycin-resistant* Enterococcus *sp.
WBC: White blood cell

## References

[1] Le ADK, Enweze L, DeBaun MR, Dragoo JL (2018). Current clinical recommendations for use of platelet-rich plasma. Curr Rev Musculoskelet Med.

[2] Ban KA, Minei JP, Laronga C, Harbrecht BG, Jensen EH, Fry DE (2017). American College of Surgeons and Surgical Infection Society: surgical site infection guidelines, 2016 update. J Am Coll Surg.

[3] Hamman BL, Stout LY, Theologes TT, Sass DM, da Graca B, Filardo G (2014). Relation between topical application of platelet-rich plasma and vancomycin and severe deep sternal wound infections after a first median sternotomy. Am J Cardiol.

[4] Patel AN, Selzman CH, Kumpati GS, McKellar SH, Bull DA (2016). Evaluation of autologous platelet rich plasma for cardiac surgery: outcome analysis of 2000 patients. J Cardiothorac Surg.

[5] Dorge H, Sellin C, Bury MC, Drescher A, Seipelt R, Grossmann M (2013). Incidence of deep sternal wound infection is not reduced with autologous platelet rich plasma in high-risk cardiac surgery patients. Thorac Cardiovasc Surg.

[6] Albanese A, Licata ME, Polizzi B, Campisi G (2013). Platelet-rich plasma (PRP) in dental and oral surgery: from the wound healing to bone regeneration. Immun Ageing.

[7] Yeaman MR (2014). Platelets: at the nexus of antimicrobial defence. Nat Rev Microbiol.

[8] Lacci KM, Dardik A (2010). Platelet-rich plasma: support for its use in wound healing. Yale J Biol Med.

[9] Frelinger AL, Torres AS, Caiafa A, Morton CA, Berny-Lang MA, Gerrits AJ (2016). Platelet-rich plasma stimulated by pulse electric fields: platelet activation, procoagulant markers, growth factor release and cell proliferation. Platelets.

[10] Yeaman MR (2010). Platelets in defense against bacterial pathogens. Cell Mol Life Sci.

[11] Xu Y, Yu H, Sun H (2014). Targeting the host hemostatic system function in bacterial infection for antimicrobial therapies. J Thromb Thrombolysis..

[12] Kuffler DP (2015). Improving the ability to eliminate wounds and pressure ulcers. Wound Repair Regen.

[13] Fabbro MD, Bortolin M, Taschieri S, Ceci C, Weinstein RL (2016). Antimicrobial properties of platelet-rich preparations A systematic review of the current pre-clinical evidence. Platelets..

[14] Mariani E, Canella V, Berlingeri A, Bielli A, Cattini L, Landini MP (2015). Leukocyte presence does not increase microbicidal activity of Platelet-rich Plasma in vitro. BMC Microbiol.

[15] Bielecki T, Ehrenfest DMD, Everts PA, Wiczowski A (2012). The role of leukocytes from L-PRP/L-PRF in wound healing and immune defense: new perspectives. Curr Pharm Biotechnol.

[16] Gonzalez AC, Costa TF, Andrade ZA, Medrado AR (2016). Wound healing—a literature review. An Bras Dermatol.

[17] Bowler PG, Davies BJ (1999). The microbiology of acute and chronic wounds. Wounds.

[18] Chicharro-Alcantara D, Rubio-Zaragoza M, Damia-Gimenez E, Carrillo-Poveda JM, Cuervo-Serrato B, Pelaez-Gorrea P (2018). Platelet rich plasma: new insights for cutaneous wound healing management. J Funct Biomater.

[19] Bowler PG, Duerden BI, Armstrong DG (2001). Wound microbiology and associated approaches to wound management. Clin Microbiol Rev.

[20] Kuffler DP (2015). Platelet-rich plasma promotes axon regeneration, wound healing, and pain reduction: fact or fiction. Mol Neurobiol.

[21] Bayer A, Lammel J, Rademacher F, Grob J, Siggelkow M, Lippross S (2016). Platelet-released growth factors induce the antimicrobial peptide human beta-defensin-2 in primary keratinocytes. Exp Dermatol.

[22] Shannon O (2017). Determining platelet activation and aggregation in response to bacteria. Methods Mol Biol.

[23] Tohidnezhad M, Varoga D, Podschun R, Wruck CJ, Seekamp A, Brandenburg L-O (2011). Thrombocytes are effectors of the innate immune system releasing human beta defensin-3. Injury.

[24] Everts PA, Hoffmann J, Weibrich G, Mahoney CB, Schonbergr PAM, van Zundert A (2006). Differences in platelet growth factor release and leucocyte kinetics during autologous platelet gel formation. Transfus Med.

[25] Aktan I, Dunkel B, Cunningham FM (2013). Equine platelets inhibit *E. coli* growth and can be activated by bacterial lipopolysaccharide and lipoteichoic acid although superoxide anion production does not occur and platelet activation is not associated with enhanced production by neutrophils. Vet Immunol Immunopathol..

[26] OCEBM Levels of Evidence Working Group. “The Oxford Levels of Evidence 2”. Oxford centre for evidence-based medicine. https://www.cebm.ox.ac.uk/resources/levels-of-evidence/ocebm-levels-of-evidence. Accessed 23 Feb 2021.

[27] Hooijmans CR, Rovers MM, de Vries RB, Leenaars M, Ritskes-Hoitinga M, Langendam MW (2014). SYRCLE's risk of bias tool for animal studies. BMC Med Res Methodol.

[28] Intravia J, Allen DA, Durant TJ, McCarthy MB, Russell R, Beitzel K, Cote MP, et al. Muscles Ligaments Tendons J. 2014;4:79–84. eCollection 2014 Jan. PMC4049655 24932452

[29] Li T, Ma Y, Wang M, Wang T, Wei J, Ren R (2019). Platelet-rich plasma plays an antibacterial, anti-inflammatory and cell proliferation-promoting role in an in vitro model for diabetic infected wounds. Infect Drug Resist.

[30] Burnouf T, Chou ML, Wu YW, Su CY, Lee LW (2013). Antimicrobial activity of platelet (PLT)-poor plasma, PLT-rich plasma, PLT gel, and solvent/detergent-treated PLT lysate biomaterials against wound bacteria. Transfusion.

[31] Rozalski MI, Micota B, Saowska B, Paszkiewicz M, Wieckoska-Szakiel M, Rozalska B (2013). Antimicrobial/anti-biofilm activity of expired blood platelets and their released products. Postepy Hig Med Dosw.

[32] Serraino GF, Dominijanni A, Jiritano F, Rossi M, Cuda A, Caroleo S (2015). Platelet-rich plasma inside the sternotomy wound reduces the incidence of sternal wound infections. Int Wound J.

[33] Knafl D, Thalhammer F, Vossen MG (2017). In-vitro release pharmacokinetics of amikacin, teicoplanin and polyhexanide in a platelet rich fibrin-layer (PRF)-a laboratory evaluation of a modern, autologous wound treatment. PLoS ONE.

[34] Bayer A, Tohidnezhad M, Berndt R, Lippross S, Behrendt P, Kluter T (2018). Platelet-released growth factors inhibit proliferation of primary keratinocytes in vitro. Ann Anat.

[35] Zhang W, Guo Y, Kuss M, Shi W, Aldrich AL, Untrauer J (2019). Platelet-rich plasma for the treatment of tissue infection: preparation and clinical evaluation. Tissue Eng Part B Rev.

[36] Lu B, Wang T, Li Z, Dai F, Lv L, Tang F (2016). Healing of skin wounds with a chitosan-gelatin sponge loaded with tannins and platelet-rich plasma. Int J Biol Macromol.

[37] Shibata M, Takagi G, Kudo M, Kurita J, Kawamoto Y, Miyagi Y (2018). Enhanced sternal healing through platelet-rich plasma and biodegradable gelatin hydrogel. Tissue Eng Part A.

[38] Bielecki TM, Gazdzik TS, Arendt J, Szczepanski T, Krol W, Wielkoszynski A (2007). Antibacterial effect of autologous platelet gel enriched with growth factors and other active substances: an in vitro study. J Bone Joint Surg Br.

[39] Moojen DJ, Everts PA, Schure M, Overdevest EP, van Zundert A, Knap JT (2008). Antimicrobial activity of platelet-leukocyte gel against *Staphylococcus aureus*. J Orthop Res.

[40] Tohidnezhad M, Varoga D, Wruck CJ, Podschun R, Sachweh BH, Bornemann J (2012). Platelets display potent antimicrobial activity and release human beta-defensin 2. Platelets.

[41] Li H, Hamza T, Tidwell JE, Clovis N, Li B (2013). Unique antimicrobial effects of platelet-rich plasma and its efficacy as a prophylaxis to prevent implant-associated spinal infection. Adv Healthc Mater.

[42] Li GY, Yin JM, Ding H, Jia WT, Zhang CQ (2013). Efficacy of leukocyte- and platelet-rich plasma gel (L-PRP gel) in treating osteomyelitis in a rabbit model. J Orthop Res.

[43] Edelblute CM, Donate AL, Hargrave BY, Heller LC (2015). Human platelet gel supernatant inactivates opportunistic wound pathogens on skin. Platelets.

[44] Cetinkaya RA, Yilmaz S, Ünlü A, Petrone P, Marini C, Karabulut E, Urkan M (2018). The efficacy of platelet-rich plasma gel in MRSA-related surgical wound infection treatment: an experimental study in an animal model. Eur J Trauma Emerg Surg.

[45] Çetinkaya RA, Yenilmez E, Petrone P, Yılmaz S, Bektöre B, Şimsek B (2019). Platelet-rich plasma as an additional therapeutic option for infected wounds with multi-drug resistant bacteria: in vitro antibacterial activity study. Eur J Trauma Emerg Surg.

[46] Cieslik-Bielecka A, Reichert P, Skowronski R, Krolikowska A, Bielecki T (2019). A new aspect of in vitro antimicrobial leukocyte- and platelet-rich plasma activity based on flow cytometry assessment. Platelets.

[47] Nimal TR, Baranwal G, Bavya MC, Biswas R, Jayakumar R (2016). Anti-staphylococcal activity of injectable nano tigecycline/chitosan-PRP composite hydrogel using Drosophila melanogaster model for infectious wounds. ACS Appl Mater Interfaces.

[48] Yassin GE, Dawoud MHS, Wasfi R, Maher A, Fayez AM (2019). Comparative lyophilized platelet-rich plasma wafer and powder for wound-healing enhancement: formulation, in vitro and in vivo studies. Drug Dev Ind Pharm.

[49] Ikono R, Mardliyati E, Agustin IT, Ulfi M, Andrianto D, Hasanah U (2018). Chitosan—PRP nanosphere as a growth factors slow releasing device with superior antibacterial capability. Biomed Phys Eng Express.

[50] Farghali HA, AbdElKader NA, AbuBakr HO, Aljuaydi SH, Khattab MS, Elhelw R (2019). Antimicrobial action of autologous platelet-rich plasma on MRSA-infected skin wounds in dogs. Sci Rep.

[51] Wozniak W, Tarnas M, Milek T, Mlosek KR, Ciostek P (2016). The effect of local platelet rich plasma therapy on the composition of bacterial flora in chronic venous leg ulcer. Pol J Microbiol.

[52] Englert SJ, Estep TH, Ellis-Stoll CC (2008). Postoperative surgical chest and leg incision sites using platelet gel: a retrospective study. J Extra Corpor Technol.

[53] Englert SJ, Estep TH, Ellis-Stoll CC (2005). Autologous platelet gel applications during cardiovascular surgery: effect on wound healing. J Extra Corpor Technol.

[54] Tran TD-X, Le PT-B, Van Pham P. Diabetic foot ulcer treatment by activated platelet rich4plasma: a clinical study. Biomed Res Ther. 2014;1:37–42.

[55] Vang SN, Brady CP, Christensen KA, Allen KR, Anderson JE, Isler JR (2007). Autologous platelet gel in coronary artery bypass grafting: effects on surgical wound healing. J Extra Corpor Technol.

[56] Singer AJ, Clark RA (1999). Cutaneous wound healing. N Engl J Med.

[57] Sears ED, Wu L, Waljee JF, Momoh AO, Zhong L, Chung KC (2016). The impact of deep sternal wound infection on mortality and resource utilization: a population-based study. World J Surg.

[58] Yeaman MR (1997). The role of platelets in antimicrobial host defense. Clin Infect Dis.

[59] Deppermann C, Kubes P (2018). Start a fire, kill the bug: the role of platelets in inflammation and infection. Innate Immun.

[60] Cieslik-Bielecka A, Glik J, Skowronski R, Bielecki T (2016). Benefit of leukocyte- and platelet-rich plasma in operative wound closure in oral and maxillofacial surgery. Biomed Res Int.

